# Large-Scale CRISPR Screen of LDLR Pathogenic Variants

**DOI:** 10.34133/research.0203

**Published:** 2023-07-25

**Authors:** Mengjing Li, Lerong Ma, Yiwu Chen, Jianing Li, Yanbing Wang, Wenni You, Hongming Yuan, Xiaochun Tang, Hongsheng Ouyang, Daxin Pang

**Affiliations:** ^1^ Key Lab for Zoonoses Research, Ministry of Education, Animal Genome Editing Technology Innovation Center, College of Animal Sciences, Jilin University, Changchun, Jilin Province 130062, China.; ^2^ The Institute of Translational Medicine, Tianjin Union Medical Center of Nankai University, Tianjin 300071, China.; ^3^ Chongqing Research Institute, Jilin University, Chongqing 401123, China.; ^4^ Chongqing Jitang Biotechnology Research Institute Co. Ltd., Chongqing, China.

## Abstract

Familial hypercholesterolemia (FH) is a frequently occurring genetic disorder that is linked to early-onset cardiovascular disease. If left untreated, patients with this condition can develop severe cardiovascular complications. Unfortunately, many patients remain undiagnosed, and even when diagnosed, the treatment is often not optimal. Although mutations in the *LDLR* gene are the primary cause of FH, predicting whether novel variants are pathogenic is not a straightforward task. Understanding the functionality of LDLR variants is crucial in uncovering the genetic basis of FH. Our study utilized CRISPR/Cas9 cytosine base editors in pooled screens to establish a novel approach for functionally assessing tens of thousands of LDLR variants on a large scale. A total of more than 100 single guide RNAs (sgRNAs) targeting *LDLR* pathogenic mutations were successfully screened with relatively high accuracy. Out of these, 5 sgRNAs were further subjected to functional verification studies, including 1 in the promoter, 1 in the antisense RNA, 1 in the exon, and 2 in the intron. Except for the variant caused by the sgRNA located at intron 16, the functionalities of the other LDLR variants were all downregulated. The high similarity of *LDLR* intron sequences may lead to some false positives. Overall, these results confirm the reliability of the large-scale screening strategy for functional analysis of LDLR variants, and the screened candidate pathogenic mutations could be used as an auxiliary means of clinical gene detection to prevent FH-induced heart disease.

## Introduction

Familial hypercholesterolemia (FH) is an autosomal dominant metabolic disease characterized by a high level of low-density lipoprotein cholesterol (LDL-c) in plasma. Sustained high levels of LDL-c in plasma lead to atherosclerosis, which, in turn, increases the risk of cardiovascular disease. If left untreated, patients can develop early-onset coronary heart disease or even die [1,2]. Despite the heavy burden of cardiovascular disease caused by FH, it is still underdiagnosed and undertreated worldwide.

Autosomal dominant mutations in the *ApoB100*, *PCSK9*, and *LDLR* genes are the main genetic causes of FH. Most FH individuals are heterozygous for mutations in one of these genes, which have an incidence rate of 1/200. Homozygosity is observed in very few patients, with an incidence of 1/300,000 [3–5]. Approximately 50% of heterozygous FH patients develop cardiovascular disease within 40 or 50 years [6]. Compared with heterozygous carriers, homozygous patients have higher plasma LDL-c levels and earlier onset of cardiovascular disease [7,8]. Defects in LDLR are the main genetic cause of FH, and approximately 85% of patients have pathogenic mutations in *LDLR*.

LDLR, a glycoprotein found in cell membranes, plays a crucial role in maintaining cholesterol homeostasis by binding to circulating LDL and facilitating its internalization as part of the LDL–LDLR complex. Deficiency in LDLR can result in the accumulation of LDL-c in the bloodstream, which increases the risk of cardiovascular disease. *LDLR* loss-of-function (LOF) mutations can be categorized into five classes based on their effects. Class 1 mutations result in defective LDLR synthesis, while class 2 mutations lead to impaired release of LDLR from the endoplasmic reticulum. Class 3 mutations affect LDL binding, class 4 mutations impact internalization of the LDL–LDLR complex, and class 5 mutations affect the recycling of LDLR back to the cell membrane [9–11].

The Leiden Open Variation Database 3 (LOVD3) has reported over 2,000 unique mutations in the *LDLR* gene that result in FH. However, only a small fraction, less than 15%, of these mutations have undergone comprehensive functional analysis [12]. Gene mutations can take various forms such as insertion mutations, deletion mutations, frameshift mutations, and point mutations (nonsense, transition, and transversion). Among these, insertion, deletion, frameshift, and nonsense mutations have been found to affect the function of LDLR protein and are therefore considered pathogenic without the need for further evidence. However, in the case of transition and transversion point mutations of *LDLR*, their pathogenicity can only be confirmed through functional studies that demonstrate their impact on LDL metabolism, as part of clinical diagnosis [13]. According to estimates, there are tens of millions of individuals with FH globally, and the incidence of FH in the general population is as high as 1 in 200. However, less than 1% of FH patients receive a diagnosis, leaving the rest to suffer from high LDL-c [14,15]. It is possible that these undiagnosed individuals have pathogenic point mutations in the *LDLR* gene, and it is crucial to diagnose and treat FH early to effectively prevent cardiovascular disease.

Large-scale genome editing offers a potential means for the functional characterization of genetic variants. Saturation mutagenesis, a method that involves CRISPR/Cas9-mediated homology-directed repair (HDR), can produce all possible single-nucleotide mutations in coding regions and noncoding sequences. In a study by Findlay et al. [16,17], the functional effects of almost 4,000 single-nucleotide mutations in the *BRCA1* gene were analyzed using this saturation mutation approach. However, CRISPR/Cas9-mediated HDR is usually inefficient, and DNA double-strand breaks (DSBs) in the target genome induced by the Cas9 protein can lead to genomic rearrangements and cell cycle arrest or cell death [18–20]. Base editor (BE) systems that combine the CRISPR/Cas9 platform with adenine deaminase or cytidine deaminase have been developed for accurate A-to-G or C-to-T conversions with high editing efficiency. These BEs are capable of introducing transition mutations at the target site in the genome without the need for a donor template, and without generating genomic DSBs [21,22]. Consequently, BE systems are a promising option for subsaturation variation screening. A recent study utilized cytosine base editor (CBE) systems in combination with pooled screening to efficiently assess the functional effects of human nucleotide mutations. Numerous LOF mutations have been identified in DNA damage repair genes [23,24]. Therefore, the availability of the BE system and pooled screening will facilitate the application of large-scale screening for human genetic variation.

Based on the CBE system and pooled screening, we established a novel strategy for large-scale screening of *LDLR* LOF mutations, obtaining hundreds of *LDLR* pathogenic point mutations, so that some potential FH patients can be diagnosed by early gene screening. We hope our functional screening will be useful for the auxiliary clinical diagnosis of FH.

## Results

### Generation of a BE4-sgRNA pool targeting the *LDLR* genome

**Fig. 1.**
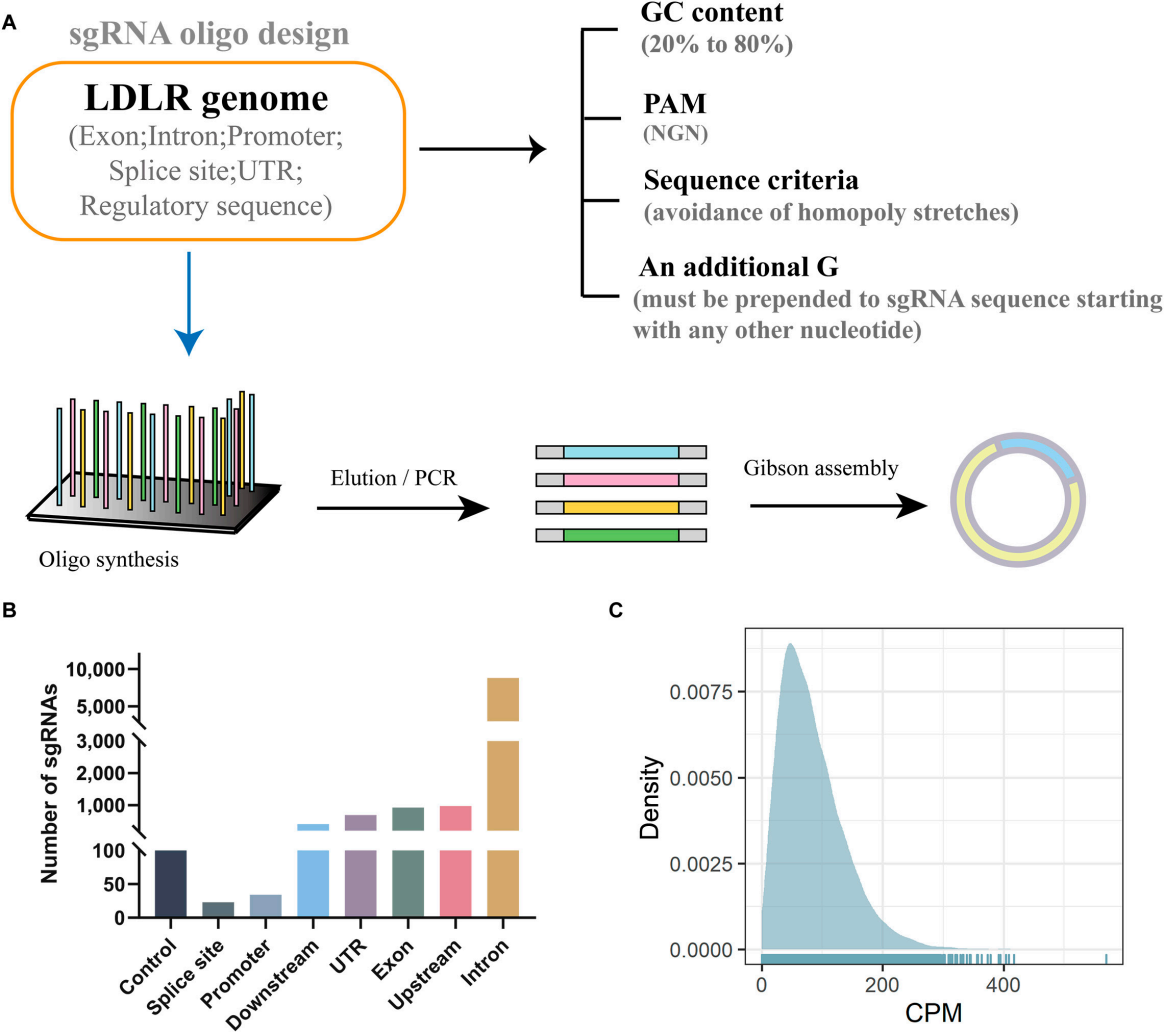
Generation of a lentiviral sgRNA library targeting *LDLR*. (A) Pipeline for designing and constructing the sgRNA libraries. (B) The number of sgRNAs designed for different regions of *LDLR*. (C) NGS of sgRNA targeting sequences in plasmid pools. CPM, counts per million.

The BE4-single guide RNA (sgRNA) library development process is illustrated in Fig. 1A. Human LDLR's whole genome sequence and regulatory sequences (5,000 bp upstream and 2,000 bp downstream of *LDLR*) were used to design sgRNAs. All NGN PAM sequences were targeted, and an editing window (13 to 18 nucleotides from the PAM) containing a C was identified. If the sgRNA sequence's first nucleotide did not start with “G”, a “G” was added at the beginning. The sgRNA sequence's %GC content was between 20 and 80 and could not contain homopolymers of more than 4 bases. After excluding duplicate sgRNAs, the BE4-sgRNA pool comprised 11,877 specific LDLR-sgRNAs and 100 control sgRNAs (Supplementary Data 1). The BE4-sgRNA pool consisted of a total of 924 sgRNAs targeting exons, 8,822 sgRNAs targeting introns, 23 sgRNAs targeting splice sites, 34 sgRNAs targeting promoters, 690 sgRNAs targeting UTRs, 974 sgRNAs targeting upstream regulatory sequences, and 410 sgRNAs targeting downstream regulatory sequences. Additionally, there were 100 control sgRNAs that did not target any human genomic loci (Fig. 1B). These sgRNAs were synthesized as an oligo array and cloned into lentiviral vectors using the Gibson Assembly method. Deep sequencing was performed to test the quality of the LDLR-BE4-sgRNA expression plasmid library. The results showed that 99.9% of sgRNAs were present in the library, and 91.7% of sgRNAs were within a 10-fold difference in frequency, indicating high uniformity throughout the library (Fig. 1C and Supplementary Data 2).

### Strategy for large-scale screening of LOF variants in *LDLR*: Construction of a de novo cholesterol synthesis-deficient cell line

**Fig. 2.**
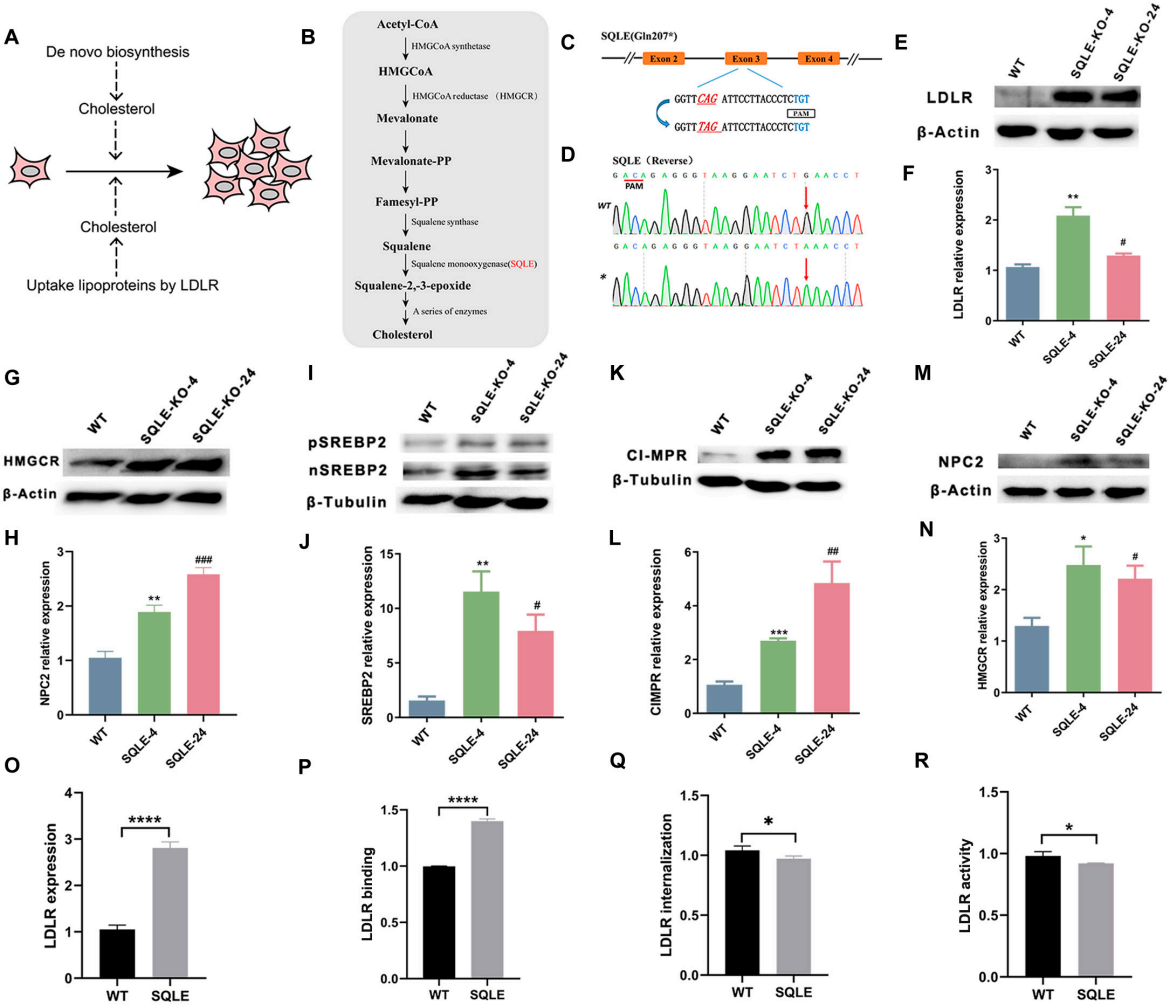
Generation of de novo cholesterol synthesis-deficient cells. (A) Sources of cholesterol in mammalian cells. (B) Steps in the de novo synthesis of intracellular cholesterol. (C) The target sequence at the *SQLE* locus. The PAM sequence, sgRNA target sequence, and substituted bases are shown in blue, black, and red, respectively. (D) Sequence chromatogram of target regions in *SQLE*. (E and F) Protein and transcript expression levels of LDLR in WT and SQLE-KO cells. (G and H) Protein and transcript expression levels of HMGCR in WT and SQLE-KO cells. (I) Analysis of SREBP2 cleavage using WT and SQLE-KO cells. pSREBP2, precursor of SREBP2; nSREBP2, nuclear form of SREBP2. (J) Transcript expression levels of SREBP2 in WT and SQLE-KO cells. (K and L) Protein and transcript expression levels of CI-MPR in WT and SQLE-KO cells. (M and N) Protein and transcript expression levels of NPC2 in WT and SQLE-KO cells. (O) LDLR expression at the cell membrane in WT and SQLE-KO cells. (P) The LDL binding ability of LDLR was measured in WT and SQLE-KO cells. (Q) Measurement of the internalization efficiency of LDLR in WT and SQLE-KO cells. (R) Measurement of LDLR activity in WT and SQLE-KO cells. Values are denoted as the mean ± SEM, 2-tailed unpaired *t* test, *P < 0.05, **P < 0.01, ***P < 0.001.

In vitro cultured mammalian cells have 2 primary sources of cholesterol. The first is de novo synthesis from acetyl coenzyme A. The second is imported from the environment through LDLR uptake (Fig. 2A and B). Both endogenous synthesis and exogenous uptake of cholesterol are essential to maintain cholesterol homeostasis. If the *LDLR* gene undergoes a loss-of-function (LOF) mutation, its ability to take up cholesterol from LDL may be impaired. However, endogenous cholesterol synthesis may compensate for this deficiency. Therefore, we hypothesize that cells with *LDLR* LOF mutation and endogenous cholesterol synthesis defects may experience an impact on their cell growth state. To test this hypothesis, we initially created cells that are deficient in endogenous cholesterol synthesis.

Squalene monooxygenase (SQLE) is a rate-limiting enzyme of cholesterol synthesis in mammalian cells, as it can catalyze the conversion of squalene to squalene-2,3-epoxide (Fig. 2B) [25]. We targeted exon 3 of *SQLE* to generate an *SQLE* gene termination mutation using the NG-AncBE4max system (Fig. 2C). The Sanger sequencing results showed that the mutation efficiency at *SQLE* loci was 36% (Fig. S1A and B). Subsequently, homozygous individual cell colonies with *SQLE* termination mutations were screened (Fig. 2D). Sequencing results revealed no off-target effects (Fig. S5). The Western blot results indicated that SQLE-null cells were incapable of expressing the SQLE protein (Fig. S1C). Furthermore, the expression of LDLR was significantly increased in SQLE-null cells compared to wild-type (WT) cells, which indicated that cells that cannot synthesize cholesterol de novo mainly depend on the uptake of LDLR (Fig. 2E and F and Fig. S1D). Additionally, the expression of HMGCR was found to be significantly higher in SQLE-null cells compared to WT cells (Fig. 2G and H and Fig. S1E). SREBP2 is a significant factor in regulating the transcription of cholesterol biosynthesis. When the level of intracellular cholesterol is low, SREBP2 releases independent nSREBP2 molecules that enter the nucleus and upregulate the expression of downstream genes, such as *LDLR* and *HMGCR*, which promote the synthesis of cholesterol [26]. Consistently, the decreased intracellular cholesterol levels in SQLE-null cells resulted in the upregulation of SREBP2 and nSREBP2 expression (Fig. 2I and J and Fig. S1F and G). In addition, the expression of NPC2 and CI-MPR, which are involved in intracellular cholesterol transport [27], was found to be increased in SQLE-null cells (Fig. 2K to N and Fig. S1H and I). These findings suggest that the loss of SQLE leads to an upregulation of key genes involved in cholesterol synthesis (*SREBP2* and *HMGCR*), cholesterol transport (*NPC2* and *CI-MPR*), and exogenous cholesterol uptake (*LDLR*) through negative feedback mechanisms.

Next, SQLE-null cells were assayed for LDLR expression, binding, internalization, and activity by flow cytometry. The results showed that the expression of LDLR was significantly higher in SQLE-null cells compared to WT cells (Fig. 2O). In terms of LDLR expression, the SQLE-null cells had approximately 140% binding efficiency and approximately 94% activity efficiency compared to control cells (Fig. 2P and R). However, the internalization of SQLE-null cells was reduced by approximately 5% compared to control cells (Fig. 2Q). These findings suggest that de novo cholesterol synthesis-deficient cells (HepG2-SQLE-KO) were successfully obtained.

### Strategy for large-scale screening of LOF variants in *LDLR*: Establishment of screening phenotype

**Fig. 3.**
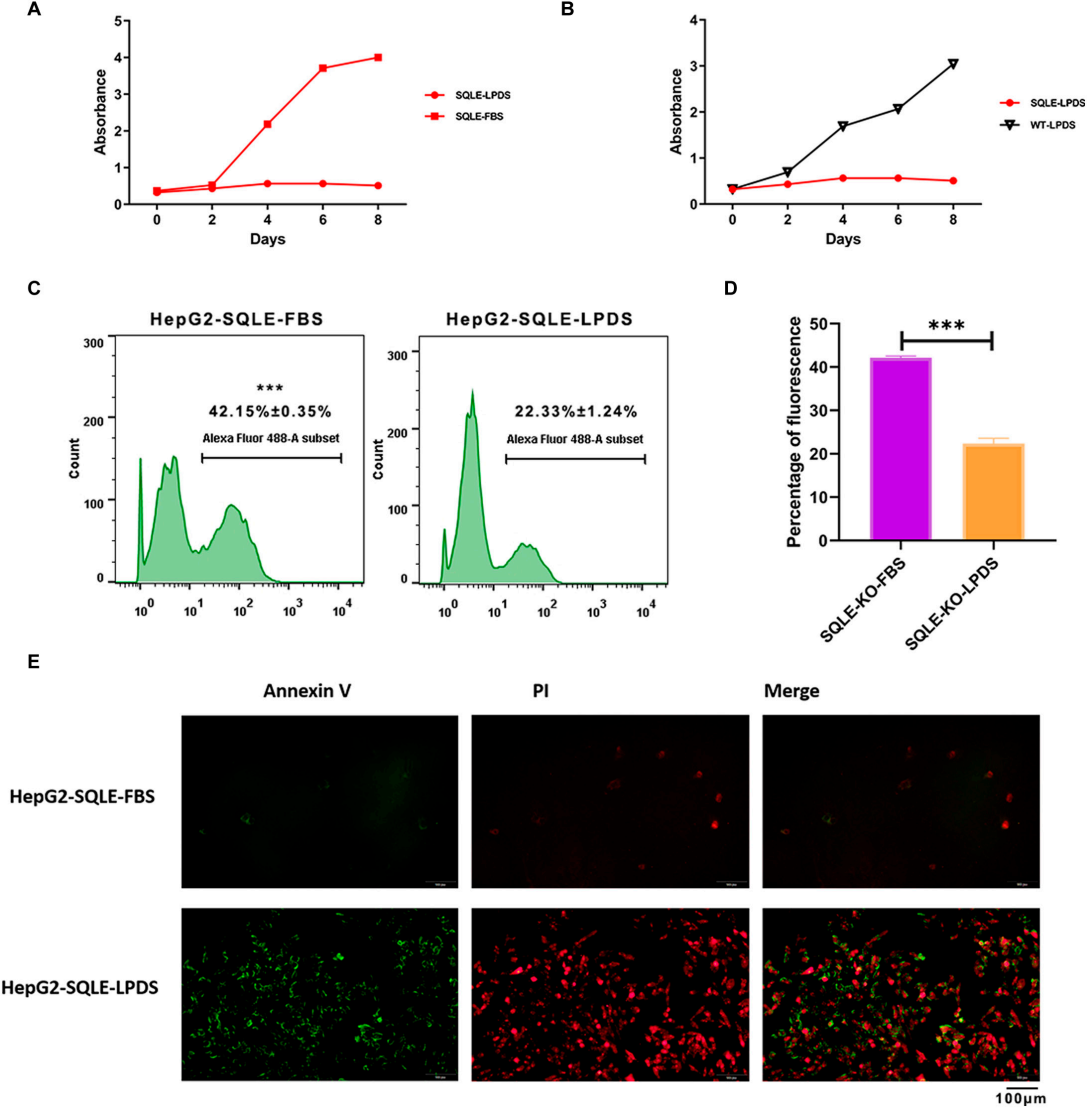
Lipoprotein depletion significantly reduced the proliferation of SQLE-KO cells. (A) SQLE-KO cells were cultured with LPDS or FBS for 8 days, and cell viability was detected by CCK8. (B) The WT and SQLE-KO cells were cultured with LPDS for 8 days, and cell viability was detected by CCK8. (C and D) Representative flow cytometry plots of the EdU proliferation assay of SQLE-KO cells that were cultured with LPDS or FBS for 4 days. Values are denoted as the mean ± SEM, 2-tailed unpaired *t* test, *P < 0.05, **P < 0.01, ***P < 0.001. (E) Apoptosis assay of SQLE-KO cells that were cultured with LPDS or FBS for 6 days. Early apoptotic cells were labeled with Annexin V–FITC, and late apoptotic cells were labeled with PI.

When cultured in lipoprotein-deficient serum (LPDS), the proliferation of HepG2-SQLE-KO cells was suppressed, and apoptosis occurred on the sixth day, while there was no significant alteration when cultured in lipoprotein-sufficient medium (FBS). In addition, WT cells did not undergo proliferation inhibition or apoptosis whenever cultured in LPDS or FBS (Fig. 3A and B and Fig. S2A). Of note, the absence of lipoproteins did not affect the proliferation of normal HepG2 cell lines, suggesting that they can obtain enough cholesterol through de novo synthesis. According to the results of the 5-ethynyl-2ʹ-deoxyuridine (EdU) proliferation assay, the proliferation of HepG2-SQLE-KO cells was significantly less than that of WT cells after being cultured in LPDS for 4 days (Fig. 3C and D). This was further confirmed by the apoptosis assay results, which showed an increase in both early and late apoptosis in HepG2-SQLE-KO cells compared to WT cells after culture in LPDS for 6 days (Fig. 3E). Consequently, if an LOF mutation occurs in *LDLR*, resulting in disordered exogenous cholesterol uptake by cells with defects in de novo cholesterol synthesis, the cells will undergo proliferation inhibition and even apoptosis. Based on this reasoning, we transduced a lentiviral LDLR-BE4-sgRNA library into HepG2-SQLE-KO cells that stably express Cas9-NG to screen for disease-causing mutations in *LDLR*.

**Fig. 4.**
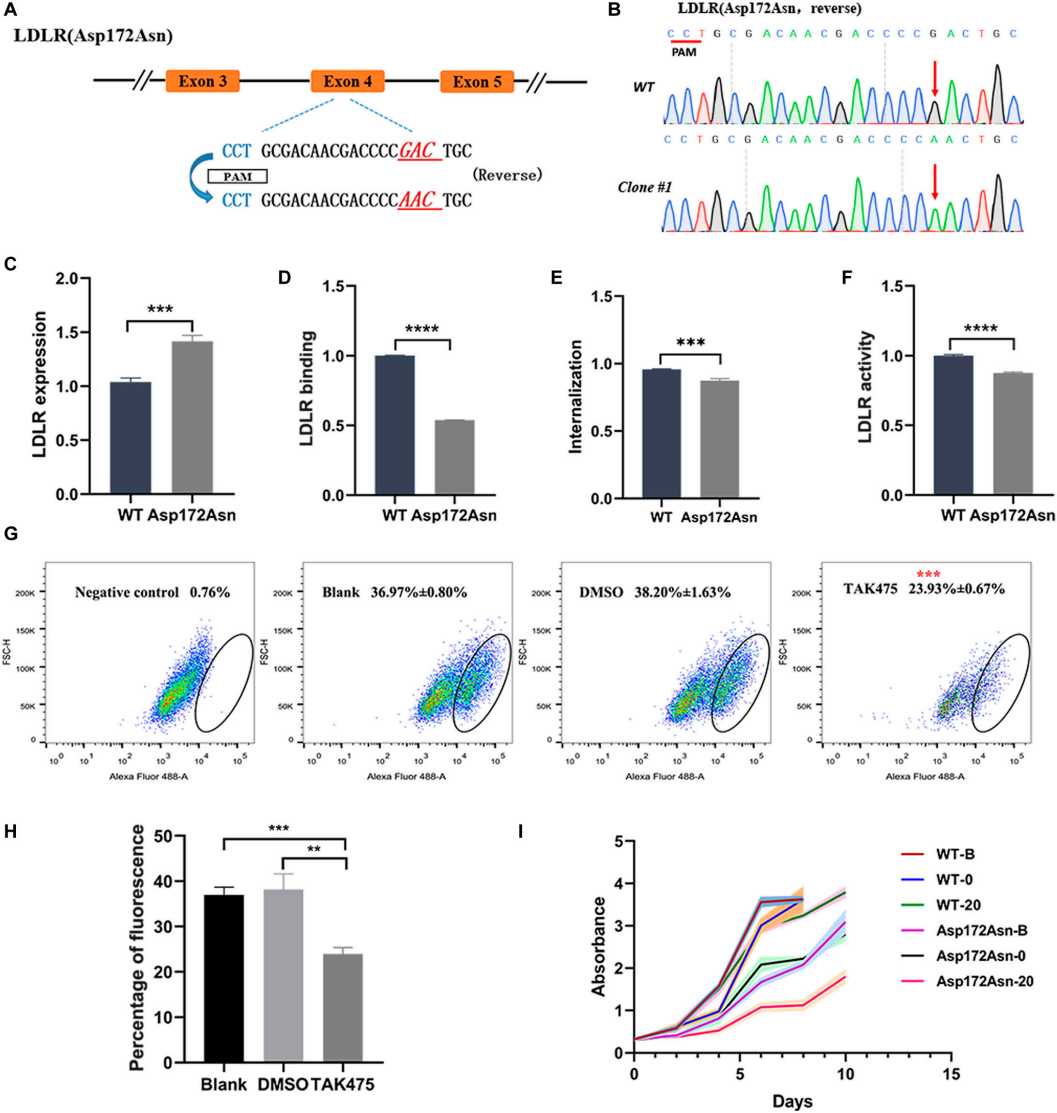
Cells with the Asp172Asn mutation in *LDLR* exhibit inhibition of proliferation in response to TAK475. (A) The target sequence at the LDLR locus. The PAM sequence, sgRNA target sequence, and substituted bases are shown in blue, black, and red, respectively. (B) Sequence chromatogram of target regions in LDLR. (C to F) The expression at the cellular membrane, binding ability, internalization efficiency, and activity of LDLR variants (Asp172Asn) compared with WT. (G and H) Representative flow cytometry plots of the EdU proliferation assay of LDLR-mutated cells that were treated with 20 μM TAK475 for 6 days. Values are denoted as the mean ± SEM, 2-tailed unpaired *t* test, *P < 0.05, **P < 0.01, ***P < 0.001. (I) *LDLR-*mutated (Asp172Asn) cells were treated with 20 μM TAK475 for 6 days, and cell viability at different time points was detected by CCK8 assay.

To investigate this phenotype further, we attempted to use NG-AncBE4max to generate homozygous cells with a known LDLR pathogenic point mutation (Asp172Asn) [28] in HepG2-SQLE-KO cells. However, we were unable to identify any homozygous cells and only heterozygous cells were obtained (Fig. 4A, Fig. S2B and C, and Table S1). This is likely due to the fact that LDLR with a homozygous pathogenic point mutation is not able to take up sufficient cholesterol to maintain cell growth in the absence of cholesterol de novo synthesis. In contrast, homozygous cells with the LDLR pathogenic point mutation (Asp172Asn) were successfully obtained in HepG2-WT cells (Fig. 4B and Table S1). The sequencing results did not reveal any off-target mutations (Fig. S6). The Asp172Asn variant of LDLR showed a 50% binding efficiency, 87% internalization efficiency, and 87% activity efficiency compared to control cells. Additionally, the LDLR expression of the Asp172Asn variant was significantly higher than that in WT cells (Fig. 4C). As a squalene synthase inhibitor [29], TAK-475 was applied to inhibit de novo cholesterol synthesis. Cells with the LDLR mutation (Asp172Asn) were treated with different concentrations (0, 0.5, 1, 4, 10, and 20 μM) of TAK-475. The CCK8 assay showed that the proliferation of cells was reduced at 20 μM TAK-475 (Fig. 4I and S2D). Additionally, the EdU proliferation assay demonstrated that cells with the LDLR mutation (Asp172Asn) had significantly lower proliferation rates after 6 days of treatment with 20 μM TAK-475 compared to WT cells (Fig. 4G and H). These findings suggest that HepG2-SQLE-KO cells can be used as a model for large-scale screening of *LDLR* disease-causing mutations.

### Identification of disease-causing mutations in *LDLR*

**Fig. 5.**
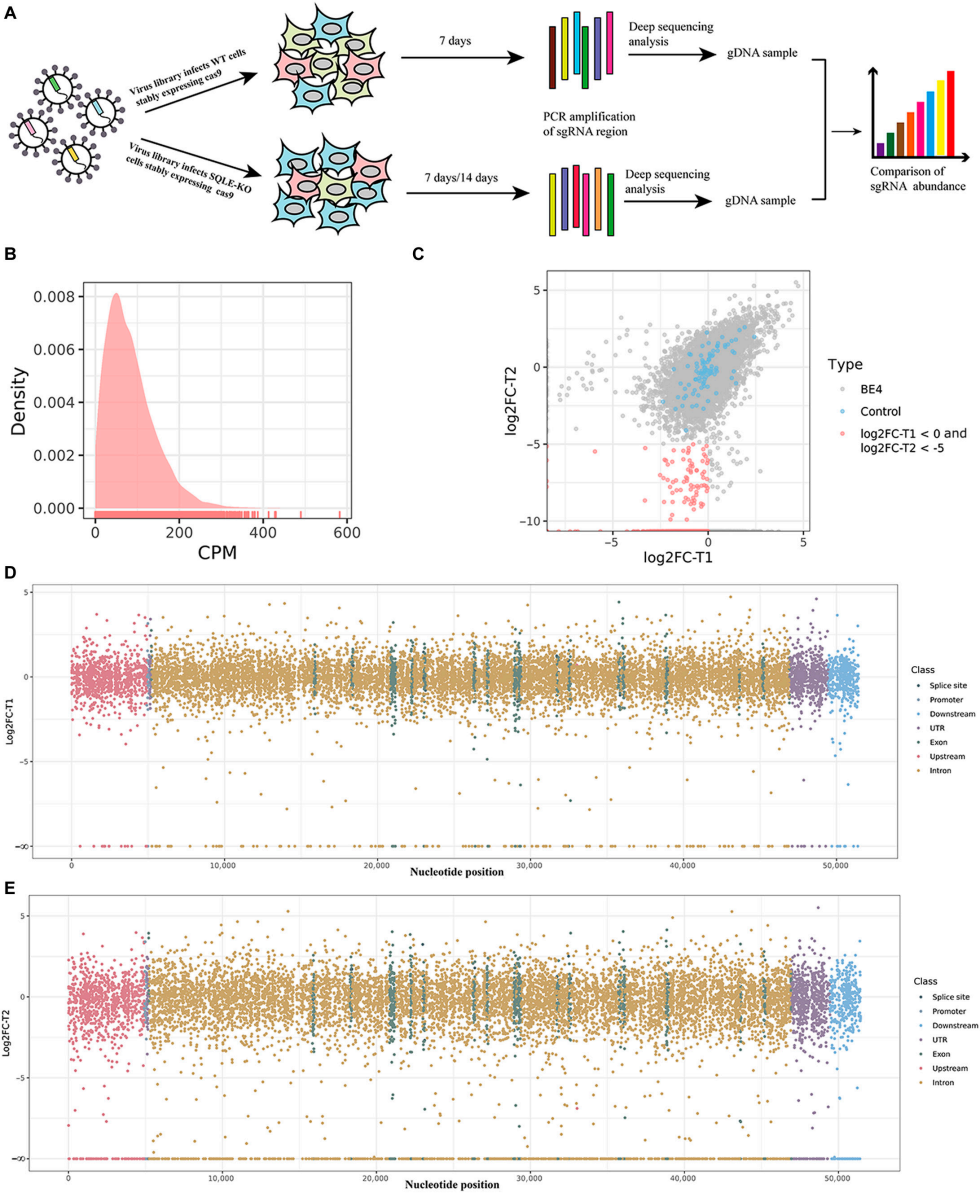
Identification of disease-causing mutations in *LDLR*. (A) Workflow and screening strategy for the BE4 screen. (B) NGS of sgRNA sequences in WT pools. CPM, counts per million. (C) Relative fold change in sgRNA sequence frequencies in the BE4-T1 sample and BE4-T2 sample compared with the WT sample. The control sgRNAs are shown in blue, the nontargeting sgRNAs are shown in gray, and sgRNAs with log2FC-T1 < 0 and log2FC-T2 < −5 are shown in red. (D and E) The log2FC-T1 or log2FC-T2 values of sgRNAs targeting different regions of *LDLR*.

The experimental procedure was illustrated in Figure S13. Initially, we created HepG2-SQLE-BE4 and HepG2-WT-BE4 cell lines that continuously expressed NG-AncBE4max with the aid of a transposon system (Figure S3A). The Western blot results validated the expression of NG-AncBE4max (Figure S3B). The functionality of NG-AncBE4max was confirmed by electrotransfection of a single sgRNA expression plasmid with no NG-AncBE4max expression plasmid into cells (Figure S3C). The LDLR-BE4-sgRNA lentivirus library was subsequently transduced into sufficient HepG2-SQLE-BE4 or HepG2-WT-BE4 cells at a multiplicity of infection (MOI) <0.3 to ensure transduction with only one sgRNA per cell. HepG2-WT-BE4-transduced cells were cultured for 7 days and used as a control, while HepG2-SQLE-BE4-transduced cells were cultured for 7 or 14 days to induce either proliferation inhibition or apoptosis due to LDLR deficiency. Next, the genomic DNA was extracted from the pooled population of cells and then amplified into fragments that contained the sgRNAs integrated into the genome (Fig. 5A). The next-generation sequencing (NGS) results demonstrated that 98.9% of sgRNAs were present in the WT library, and 91.7% of sgRNAs were present within a 10-fold difference in frequency (Fig. 5B and Supplementary Data 3).

In our experimental design, we aimed to analyze the abundance of sgRNAs targeting *LDLR* LOF mutations. To achieve this, we calculated the log2-fold changes in abundance of all sgRNAs at different time points during the culture period. This calculation provided insights into the effects of sgRNAs on LDLR function. We named the log2-fold change of sgRNAs in WT and BE4-T1 (7 days) samples as log2FC-T1 and the log2-fold change of sgRNAs in WT and BE4-T2 (14 days) samples as log2FC-T2 (Fig. 5C). The log2FC-T1 and log2FC-T2 values for each sgRNA-targeted *LDLR* genome are displayed in Fig. 5E and F and Supplementary Data 4. The majority of sgRNAs, including the control sgRNAs, had comparable scores across various samples, as anticipated. Depleted sgRNAs were defined as having log2FC-T1 < 0 and log2FC-T2 < −5, based on the log2FC-T1 and log2FC-T2 values of the control and nontarget sgRNAs (Fig. 5C). For improved accuracy, sgRNAs with log2FC-T1 < −2 and log2FC-T1 < −5 were identified as corresponding to *LDLR* LOF mutations. Notably, the *LDLR* gene's upstream regulatory sequences, downstream regulatory sequences, and intron sequences are highly similar, particularly the intron sequences. This similarity among sgRNAs targeting *LDLR* can cause off-target effects (Supplementary Data 5). This suggested that the screened sgRNAs targeting intron sequences with decreased abundance are not completely implausible for loss of LDLR function. Therefore, the candidate sgRNAs targeting the LDLR LOF mutations were excluded from analysis if their similarity to other sgRNAs in the library exceeded 17 bases. As the exon sequence and promoter sequence are highly specific, we considered the screened sgRNAs that targeted the *LDLR* promoter and exon sequence with reduced abundance to be highly reliable. In total, we screened 125 candidate sgRNAs that targeted *LDLR* LOF mutations, including 1 in the promoter (1/34 = 2.9%), 10 in the UTR (10/690 = 1.4%), 7 in the upstream regulatory sequence (7/974 = 0.7%), 3 in the downstream regulatory sequence (3/410 = 0.7%), 15 in exons (15/924 = 1.6%), and 89 in introns (89/8,822 = 1.0%) (Supplementary Data 6).

### Comparison of screened disease-causing mutations with mutation sites in the LOVD3 database

The LOVD3 has reported a total of 2,210 unique mutations in *LDLR*. Among these, 1,546 are point mutations (nonsense, transition, and transversion), while the remaining 664 are other types of mutations (insertion, deletion, and frameshift) (Fig. S12A). Out of 1,546 point mutations, only 150 were identified as pathogenic. These mutations included 226 C>T, 347 G>A, 57 A>C, 113 A>G, 51 A>T, 105 C>A, 101 C>G, 97 G>C, 158 G>T, 59 T>A, and 79 T>G mutations (Fig. S12B). Our study utilized the CBE system to perform C>T conversions on the plus and minus strands of the *LDLR* gene, which led us to select C>T and G>A mutations for analysis. Among the 226 C>T mutations, only 33 were identified as pathogenic, 48 were likely pathogenic, 41 were benign or likely benign, and 104 were uncertain (Fig. S12C). Out of the 347 reported G>A mutations, only 39 have been confirmed as pathogenic, while 153 are likely to be pathogenic, 25 are benign or likely benign, and 83 remain uncertain (Fig. S12D). In our study, the 125 candidate disease-causing sgRNAs corresponded to 224 point mutations, among which 6 point mutations were found in the LOVD database (273 pathogenic or likely pathogenic mutations and 149 benign or likely benign mutations). The corresponding 6 mutations on hg19 are located at positions g.11216124C>T, g.11216213C>T, g.11227549C>T, g.11218077G>A, g.11221436G>A, and g.11238637G>A. Out of these, g.11216124C>T, g.11216213C>T, g.11227549C>T, g.11218077G>A, and g.11221436G>A have been classified as pathogenic or likely pathogenic in the LOVD database, while g.11238637G>A has been classified as benign or likely benign (Fig. S12E and F and Supplementary Data 6). This indicates that our large-scale screening is reliable, as 5 out of 6 of the mutations correspond to pathogenic or likely pathogenic entries in the LOVD database.

### Functional study of disease-causing mutations in *LDLR*

**Fig. 6.**
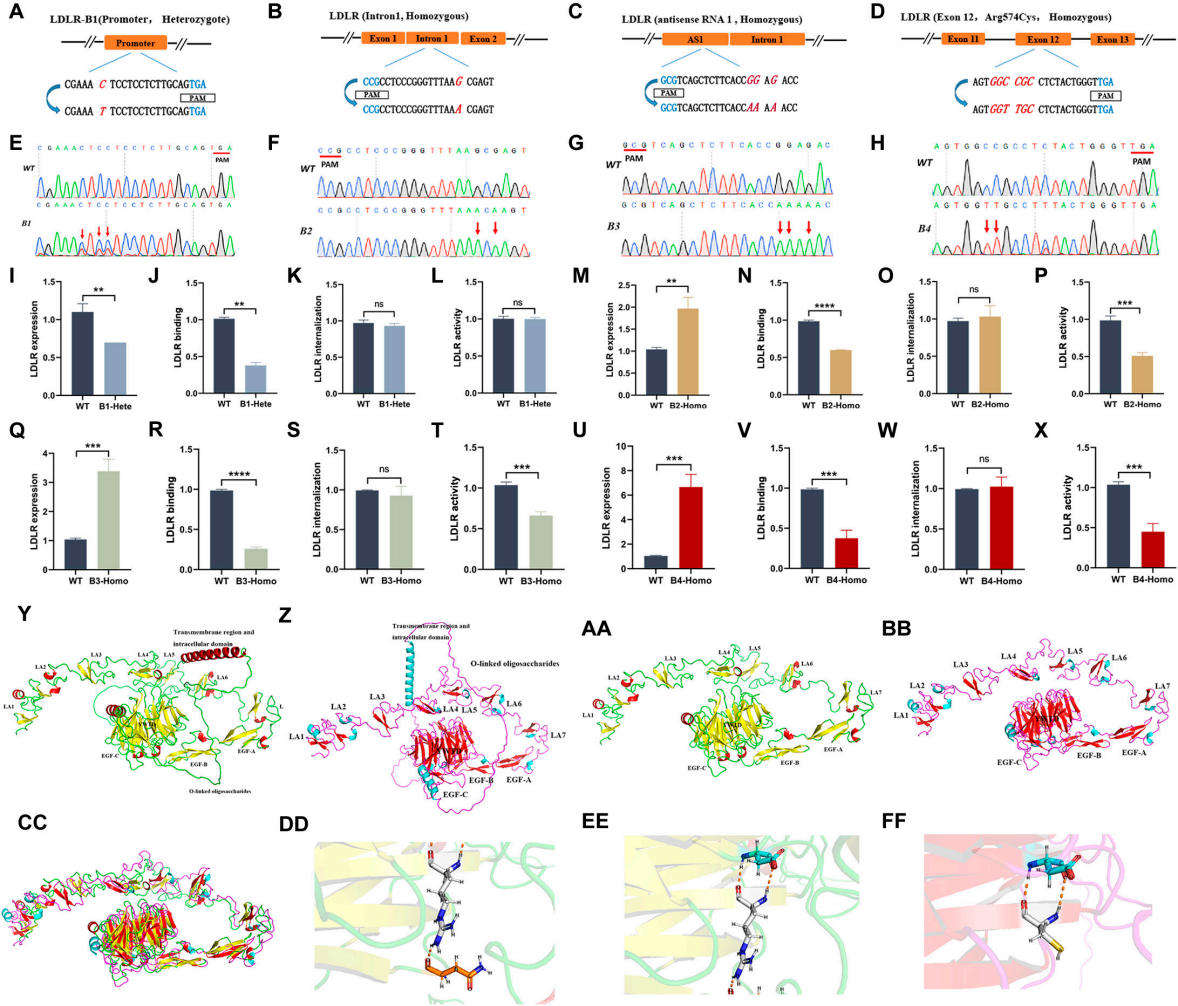
Functional characterization of LDLR variants. (A to D) The target sequences in the *LDLR* promoter, intron 1, antisense RNA, and exon 12 locus. The PAM sequence, sgRNA sequence, and substituted bases are shown in blue, black, and red, respectively. (E to H) Sequence chromatograms of target regions in the *LDLR* promoter, intron 1, antisense RNA, and exon 12 locus. (I to L) Comparison of LDLR expression, binding ability, internalization efficiency, and activity between LDLR promoter heterozygous mutant cells and WT cells. (M to P) Comparison of LDLR expression, binding ability, internalization efficiency, and activity between LDLR intron 1 homozygous mutant cells and WT cells. (O to T) Comparison of LDLR expression, binding ability, internalization efficiency, and activity between LDLR antisense RNA homozygous mutant cells and WT cells. (U to X) Comparison of LDLR expression, binding ability, internalization efficiency, and activity between LDLR exon 12 homozygous mutant cells and WT cells. Values are denoted as the mean ± SEM, 2-tailed unpaired *t* test, *P < 0.05, **P < 0.01, ***P < 0.001. (Y) Protein 3D structures of WT LDLR predicted by AlphaFold2. α-Helix, β-sheet, and random coil structures are shown in red, yellow, and green, respectively. The LA repeat domain, EGF domain, YWTD domain, O-linked oligosaccharides, transmembrane region, and intracellular domain are indicated. (Z) Protein 3D structures of an LDLR variant (p.R574C) predicted by AlphaFold2. α-Helix, β-sheet, and random coil structures are shown in blue, red, and purple, respectively (AA and BB). 3D protein structure of the LDLR ectodomain from the first cysteine-rich repeat to the EGF domain. (CC) Alignment of extracellular domains of WT LDLR and mutant (p.R574C) LDLR. (DD and EE) Arg574 is hydrogen-bonded to Asn594 and Asp569 in the WT LDLR protein structure. Hydrogen bonds are shown in orange. The C atoms, H atoms, N atoms, O atoms, and S atoms of arginine side chains are colored gray, gray, dark blue, red, and orange, respectively. The C atoms, H atoms, N atoms, O atoms, and S atoms of asparagine side chains are colored gray, gray, dark blue, red, and orange, respectively. The C atoms, H atoms, N atoms, O atoms, and S atoms of arginine side chains are colored orange, gray, dark blue, red, and orange, respectively. The C atoms, H atoms, N atoms, O atoms, and S atoms of aspartate side chains are colored light blue, gray, dark blue, red, and orange, respectively. (FF) Cys574 is hydrogen-bonded to Asp569 in the mutant LDLR protein structure. Hydrogen bonds are shown in orange. The C atoms, H atoms, N atoms, O atoms, and S atoms of cysteine side chains are colored gray, gray, dark blue, red, and orange, respectively. This figure was prepared with PyMOL.

It is not advisable to draw definitive conclusions based only on the results of primary screening because sgRNAs may be exhausted due to reproducible but unanticipated effects, such as bystander effects or off-target effects. Thus, 5 sgRNAs located in the promoter, intron 1, upstream regulatory sequence, exon 12, and intron 16 were selected for validation (Fig. 6A and D and Fig. S4A). HepG2-WT-BE4 cells continuously expressing NG-AncBE4max were transfected with individual sgRNAs and selected for cell colonies with point mutations. Polymerase chain reaction (PCR) amplification of the genomic region surrounding the editing site revealed cell colonies with heterozygous mutations in the promoter (B1), homozygous mutations in intron 1 (B2), an upstream regulatory sequence (B3), exon 12 (B4), and intron 16 (B5) (Fig. 6E to H and Fig. S4B). Meanwhile, unedited individual cell colonies were treated as WT cells. The sequencing results revealed no off-target effects of the 5 sgRNAs (Figs. S7 to S11).

Furthermore, individual cell colonies of B1 to B5 were assayed for LDLR expression, binding, internalization, and activity by flow cytometry. The results showed that the heterozygous mutation in the promoter led to a decrease in LDLR expression and binding ability. However, there were no significant changes observed in LDLR internalization and activity, which may be attributed to compensatory effects in the heterozygous cells (Fig. 6I to L). The cell colonies of B2 that had a homozygous mutation in intron 1 showed a decrease in both LDLR binding and activity, but an increase in LDLR expression. This could be attributed to the reduced function of LDLR (Fig. 6M to P). The sgRNA targeting the upstream regulatory sequence of *LDLR* was chosen randomly. Upon sequence alignment, it was discovered that the mutation was located on the antisense RNA sequence of *LDLR* [30]. The homozygous mutation in the antisense RNA led to an increase in LDLR expression but a decrease in its binding capacity and activity (Fig. 6Q to T). In comparison with WT cells, B4 individual cell colonies showed an upregulation in the expression of LDLR, while the binding and activity were downregulated (Fig. 6U to X). Homozygous mutations in exon 12 might affect the YWTD domain of LDLR, which, in turn, could affect the binding and cycling functions of LDLR. It is important to note that the homozygous mutation in intron 16 did not affect LDLR activity, indicating a false positive in the primary screen (Fig. S4C). The results of 4/5 sgRNAs were consistent with the primary screen.

AlphaFold2 can predict a protein’s 3-dimensional (3D) structure from its amino acid sequence and regularly achieves accuracy competitive with that of experimental approaches. A C-T homozygous mutation in exon 12 converts arginine to cysteine at position 574 of the LDLR protein. To assess the effects of this mutation on the protein structure, we used AlphaFold2 to predict the structures of both the WT LDLR and the mutant LDLR (Fig. 6Y and Z). Previous studies have utilized x-ray crystallography methods to determine the structure of the extracellular domain of LDLR [31]. However, the full structures of both the extracellular and intracellular domains of LDLR remained unknown. Protein 3D structure comparison revealed that the extracellular domains of both the WT and mutant LDLR are highly similar (Fig. 6AA to CC). Within the WT protein structure, Arg574 forms hydrogen bonds with Asp569 and Asn594; however, in the p.R574C variant, these hydrogen bonds with Asn594 are lost due to the cysteine substitution (Fig. 6DD to FF). This substitution is believed to destabilize the active site, ultimately leading to impaired LDLR function.

## Discussion

*LDLR* genetic variations are a known cause of FH, which is associated with an increased risk of heart attack. Clinical genomics testing using high-throughput sequencing can identify genomic variants of uncertain significance in individuals suspected of having FH. However, while many *LDLR* point mutations have been identified, most have not been functionally validated, making their causal relationship to the disease unclear. These ambiguities in interpretation hinder the clinical application of sequencing results for early prevention of FH. In this study, we utilized CRISPR-mediated cytosine base editing and large-scale pooled screen technology to investigate nucleotide variants of human *LDLR*. The modular nature of BE screening makes it particularly advantageous for pooling screening approaches [24,32].

Cholesterol in mammalian cells is derived mainly from endogenous de novo synthesis and LDLR-dependent uptake from the environment. Deficiency in LDLR function alone does not affect cell proliferation rates (Fig. 4) and thus is not suitable as a phenotype for large-scale screening of *LDLR* LOF mutations. However, in cells with defective de novo cholesterol synthesis, deficiency in LDLR inhibits cell proliferation and even induces apoptosis. Based on this, we established a new strategy to introduce and functionally assess tens of thousands of *LDLR* variants. Ultimately, more than 100 sgRNAs that are capable of causing LDLR dysfunction were identified. Further functional verification of selected *LDLR* variants showed that the homogeneous mutation targeting intron 16 did not affect the function of LDLR, whereas mutations targeting intron 1, the promoter sequence, antisense RNA1, and exon 12 negatively impacted the function of LDLR. One sgRNA did not replicate the results of the primary screen, indicating that there is a certain probability of false positives, which may be due to the high similarity of *LDLR* introns and the inevitable bystander and off-target effects of the BE4 system. Indeed, editing outside the canonical window occurred. Nevertheless, 4 of the 5 sgRNAs yielded the expected phenotype, confirming the accuracy and feasibility of the primary large-scale screening.

In summary, using this BE4 screen to functionally profile *LDLR* variants is a flexible, scalable approach, and we established a new strategy to functionally evaluate tens of thousands of *LDLR* variants. More than 100 candidate sgRNAs targeting pathogenic point mutations of *LDLR* were successfully screened with relatively high accuracy. Our findings suggest that this functional evaluation of *LDLR* variants will be useful in clinical auxiliary diagnosis of FH and could improve the utility of genetic testing in preventing cardiovascular disease risk associated with FH.

## Materials and Methods

### Plasmid construction

The blasticidin resistance gene fragment was cloned into the PiggBac transposon plasmid (pB513B) through the *NheI* and *HpaI* restriction enzyme sites, and the resulting plasmid was designated pB513B-BSD. The cytosine deaminase, nCas9-NG, and UGI fragments amplified from the NG-AncBE4max plasmid were cloned into pB513B-BSD, and the reconstructed plasmid was named pB513B-BSD-BE4. The NG-AncBE4max plasmid was maintained in our laboratory [33]. Human SQLE-specific and LDLR-specific sgRNA fragments were cloned into empty pBluescriptSKII+U6-sgRNA(F+E) vectors to produce a functional sgRNA expression vector.

### Cell electroporation and screening

Approximately 20 μg of SQLE-specific sgRNA expression plasmid and 20 μg of NG-AncBE4max plasmid were cotransfected into 3 × 10^6^ HepG2 cells using the Neon^TM^ transfection system. The electroporation conditions for the HepG2 cells were as follows: 1,280 V, 30 ms, and 1 pulse. After 2 days, the cells were seeded into 100-mm dishes to form individual cell colonies. Forty-two individual cell colonies were picked to identify C-T termination mutation events.

Approximately 3 × 10^6^ SQLE gene termination mutation homozygous cells and WT cells were electrotransfected with 300 ng of pB513B-BSD-BE4 plasmid and 10 μg of Super PiggyBac Transposase plasmid. After 3 days of blasticidin selection, the cells were seeded in 100-mm dishes to retrieve individual cell colonies. The protein expression level of NG-AncBE4max was assessed by Western blotting. The mutation efficiency of NG-AncBE4max was assessed by electrotransfection of an sgRNA construct targeting the human *FANCF* gene.

### Lentiviral sgRNA library construction

The oligonucleotides of 14,614 LDLR-sgRNAs and 100 control sgRNAs containing the homologous sequences of the lentiGuide-Puro vector were synthesized on CustomArray 90K arrays (CustomArray Inc.) and then amplified. The PCR products were cloned into the lentiGuide-Puro vector through Gibson Assembly. Gibson Assembly ligation mixtures were transformed into DH5α chemically competent cells and then cultured in liquid culture for 16 to 18 h. The extracted plasmids formed the sgRNA expression plasmid library, and sgRNA coverage and distribution were analyzed by NGS. The primers for amplification are listed in Supplementary Table S2.

### sgRNA library lentivirus production and transduction

The psPAX2 plasmid, pMD2.G plasmid, and sgRNA library plasmid were cotransfected into 293T cells using PEI transfection reagent. After 72 h of transfection, the cell supernatant containing the virus was collected and filtered with a 0.45-μm filter. Finally, the supernatant was centrifuged at 24,000 rpm, and the virus precipitate was resuspended in phosphate buffer saline (PBS).

### sgRNA library lentivirus transduction and pooled screening

Approximately 3 × 10^7^ HepG2-SQLE-BE4 or HepG2-WT-BE4 cells in 150-mm cell culture dishes were transduced with sgRNA library lentivirus at an MOI < 0.3 in the presence of 8 μg/ml polybrene. After 24 h, the virus medium was replaced with fresh medium. Puromycin was added after 48 h, and the cell genomic DNA was extracted after 7 or 14 days of screening. The genomic DNA was used as a template for amplification of the sgRNA inserts, and the amplified sgRNA fragments were then used as templates for library construction with the NEBNextUltra^TM^ DNA Library Prep kit. The resulting libraries were analyzed via NGS. The PCR amplification primer sequences used are listed in Supplementary Table S2. The NGS data were analyzed using R language as follows: (a) the reads of each sgRNA in WT and BE4-T1 and BE4-T2 samples were calculated; (b) duplicate sgRNA sequences were excluded; (c) sgRNAs with fewer than 20 reads in WT were removed; (d) the standardized count of each sgRNA in WT and BE4-T1 and BE4-T2 samples was calculated with the following formula: (sgRNA reads/total reads of sample) × 100,000,0; (e) log2 (sgRNA reads/total reads of sample) × 100,000,0 was calculated; (f) log2 (fold change) of WT and BE4-T1 of all sgRNAs and log2 (fold change) of WT and BE4-T2 of all sgRNAs were calculated; and (g) sgRNAs with log2 (fold change) of WT and BE4-T1 < −2 and log2 (fold change) of WT and BE4-T2 < −5 were considered disease-causing sgRNAs.

### Transcriptional analysis

Total RNA was extracted and then used to generate cDNA with a FastQuant RT kit (Tiangen, Beijing, China). The cDNA was used for real-time PCR to detect LDLR and SQLE transcription levels.

### Western blot analysis

Total protein was extracted by NP40 lysis buffer (Boster, Wuhan, China) and then separated by sodium dodecyl sulfate polyacrylamide gel electrophoresis (SDS-PAGE). The separated protein was transferred to polyvinylidene difluoride membranes and blocked with 5% bovine serum albumin (BSA) buffer. The BSA-blocked membranes were incubated separately with anti-SQLE (Proteintech, Wuhan, China), anti-LDLR (Proteintech, Wuhan, China), anti-HMGCR (Abcam, Cambridge, UK), anti-SREBP2 (Abcam, Cambridge, UK), anti-NPC2 (Proteintech, Wuhan, China), anti-CIMPR (Proteintech, Wuhan, China), and anti-Cas9 (Abcam, Cambridge, UK) antibodies and then incubated with secondary antibodies. The immunoblots were detected with ECL chemiluminescence substrate (Boster, Wuhan, China).

### Cell proliferation assays

For CCK8 analysis, 1 × 10^3^ cells were seeded into 96-well plates, and at 5 different time points (0, 2, 4, 6, and 8 days), CCK8 reagent (Boster, Wuhan, China) was added for 2 h. Then, the absorbance at 450 nm was measured. At least 3 replicates were analyzed for each sample.

For EdU proliferation analysis, 1 × 10^5^ cells were seeded into 6-well plates. When the cell confluence reached 70% to 80%, 50 μM EdU (RIBBIO, Guangzhou, China) was added to the medium, and the cells were incubated for 2 h. After incubation, the cells were fixed with 4% paraformaldehyde solution and then washed 3 times. The fixed cells were labeled with Apollo488 and analyzed by flow cytometry.

### Annexin V-FITC/PI assay

For the analysis of cell apoptosis, 1 × 10^4^ cells were seeded into 24-well plates. When the cell confluence reached 60% to 70%, 5 μl of Annexin V-fluorescein isothiocyanate (Annexin V-FITC) was added, and the cells were incubated for 15 min at room temperature and then observed by fluorescence microscopy. Subsequently, 5 μl of PI was added, and the cells were incubated for 5 min at room temperature in the dark and observed under a fluorescence microscope (Olympus).

### Quantification of LDLR expression, binding, internalization, and activity by flow cytometry

For the analysis of LDLR expression, cells cultured in 6-well plates were digested and incubated with human monoclonal LDLR antibody (Proteintech, Wuhan, China) for 30 min at room temperature. After 2 washes with PBS, the cells were incubated with FITC-labeled goat anti-rabbit secondary antibodies. Afterward, the cells were washed twice, and the average fluorescence intensity of FITC was analyzed by flow cytometry.

For the analysis of LDLR binding, cells cultured in 6-well plates were incubated with 20 μg/ml Dil-LDL (Yiyuan Biotechnology, Guangzhou, China) at 4 °C for 4 h. After 2 washes with PBS, the cells were fixed with 4% paraformaldehyde solution. Afterward, the cells were washed twice, and the fluorescence intensity of Dil was analyzed by flow cytometry.

For analysis of LDLR internalization and activity, cells cultured in 6-well plates were incubated with 20 μg/ml Dil-LDL at 37 °C for 4 h. After 2 washes with PBS, half of the cells were used for flow cytometry to detect total Dil fluorescence intensity, which is the activity of LDLR. The remaining half of the cells were taken to analyze LDLR internalization. Trypan blue solution (Boster, Wuhan, China) at a final concentration of 0.2% was added to the cells to eliminate extracellular signals from the noninternalized LDLR–LDL complexes. After Trypan blue was incubated with cells for 3 min, the cells were washed twice with PBS, and then the Dil fluorescence intensity was analyzed by flow cytometry. The internalization rate of LDLR can be calculated by the following formula: intracellular Dil fluorescence intensity/total Dil fluorescence intensity.

### Off-target analysis

All potential off-target sites were analyzed by Sanger sequencing. PCR products spanning the potential off-target sites were purified and sequenced. The off-target primers used are listed in Supplementary Table S2.

### Statistical analysis

Statistical analysis was performed by 2-tailed unpaired *t* test, and *P* < 0.05 was considered to be significant.

## Data Availability

All data needed to evaluate the conclusions in the paper are presented in the paper and the Supplementary Materials. Additional data related to this paper may be requested from the authors.
