# Supplementary material for: Large-Scale CRISPR Screen of LDLR Pathogenic Variants
Source: Research (Wash D C). 2023 Jul 25;6:0203. doi: 10.34133/research.0203 (PMC10368174; doi:10.34133/research.0203)
Supplement: Supplementary 1 — Figs. S1 to S13 Tables S1 and S2 Supplementary Data 1 to 6 [file research.0203.f1.zip › Supplementary Manuscript.docx]

**Large-scale CRISPR Screen of LDLR Pathogenic Variants**

Mengjing Li^1,2‡^, Lerong Ma^1,3,4‡^, Yiwu Chen^1,3,4‡^, Jianing Li^1^, Yanbing Wang^1^, Wenni You^1^, Hongming Yuan^1,3,4^, Xiaochun Tang^1,3,4^*＆Hongsheng Ouyang^1,3,4^*＆Daxin Pang^1,3,4^*

^1^ Key Lab for Zoonoses Research, Ministry of Education, Animal Genome Editing Technology Innovation Center, College of Animal Sciences, Jilin University, Changchun, Jilin Province 130062, China.

^2^ The Institute of Translational Medicine, Tianjin Union Medical Center of Nankai University, Tianjin 300071, China.

^3^ Chongqing Research Institute, Jilin University, Chongqing 401123, China.

^4^ Chongqing Jitang Biotechnology Research Institute Co., Ltd, Chongqing, China.

‡These authors contributed equally to this work.

*Corresponding author: Xiaochun Tang＆Hongsheng Ouyang＆Daxin Pang

E-mail: [xiaochuntang@jlu.edu.cn](mailto:xiaochuntang@jlu.edu.cn); [ouyh@jlu.edu.cn](mailto:ouyh@jlu.edu.cn); [pdx@jlu.edu.cn](mailto:pdx@jlu.edu.cn)


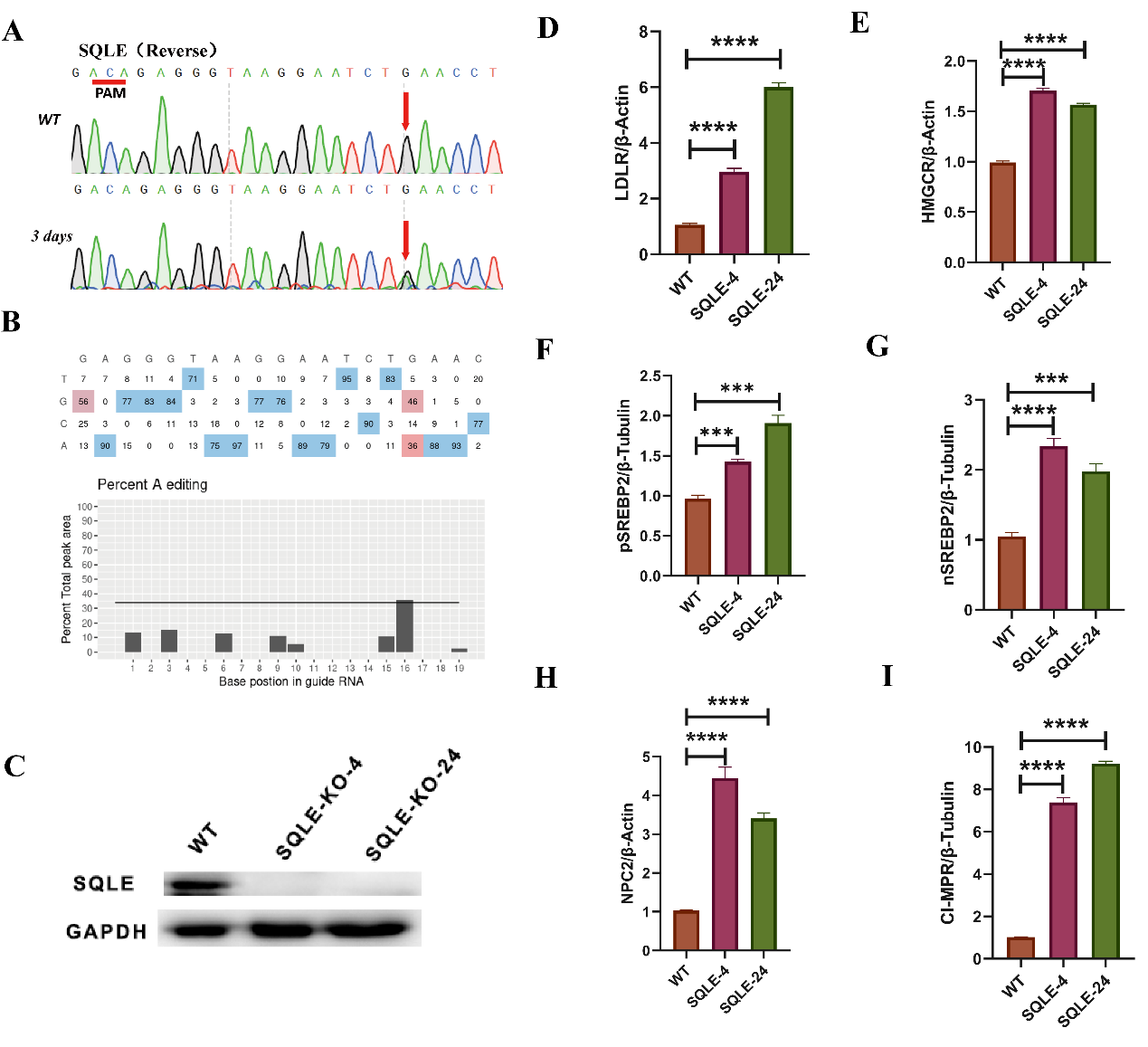


**Figure S1:** (A) The target sequence at the *HMGCR* locus. The PAM sequence, sgRNA target sequence, and substituted base are shown in orange, black, and green, respectively. (B) Sequence chromatogram of target regions in *HMGCR*. (C) Sequence chromatogram of target regions in *SQLE*. (D and E). The mutation efficiency of *HMGCR* and *SQLE* locus was predicted with the online tool EditR (https://moriaritylab.shinyapps.io/editr-master). (F) The protein expression levels of SQLE in WT and SQLE-KO cells. (G-L) Gray value analysis of LDLR, HMGCR, pSREBP2, nSREBP2, NPC2 and CI-MPR proteins in WT and SQLE-null cells.


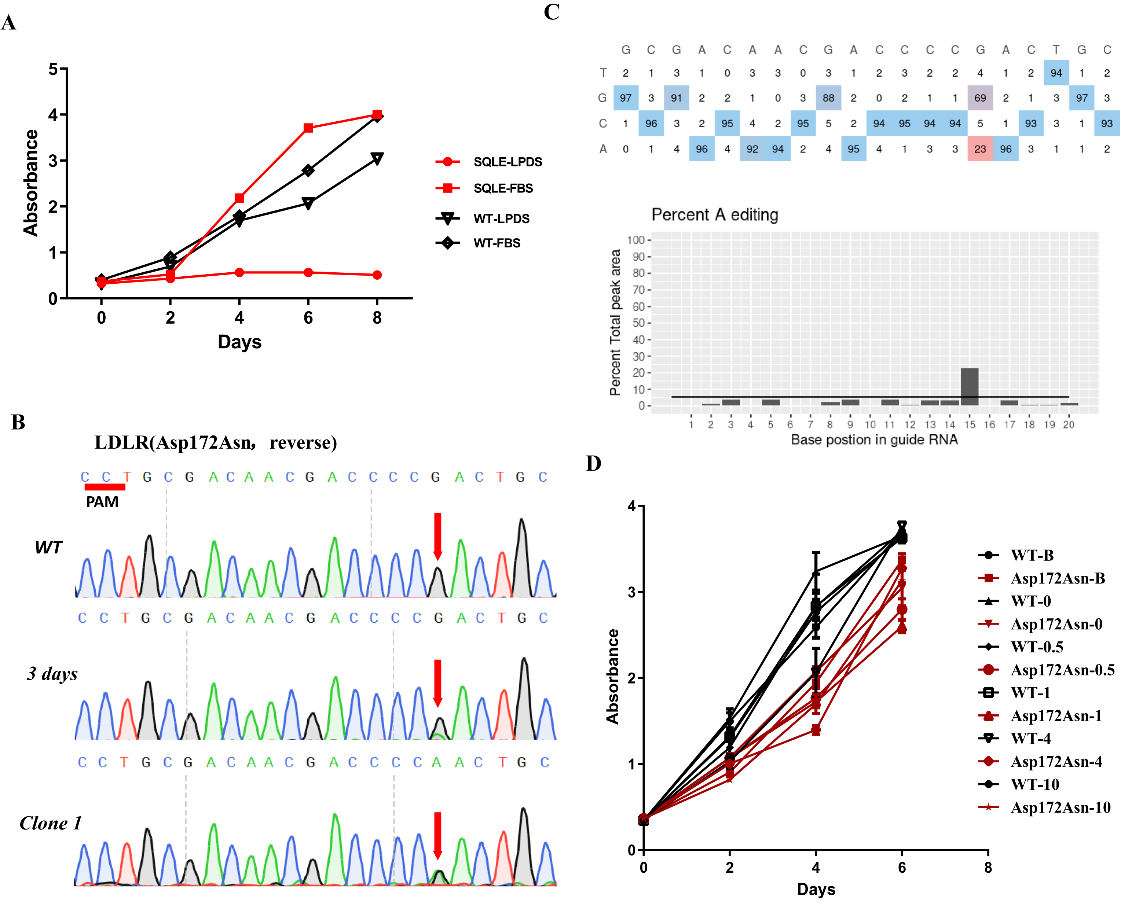


**Figure S2:** (A) SQLE-KO individual cell clones were cultured with LPDS or FBS for 8 days, and cell viability was detected by CCK8. (B) Sequence chromatogram of target regions in *LDLR*. (C) The mutation efficiency of the *LDLR* locus was predicted with the online tool EditR. (D) The cells with *LDLR* mutation (Asp172Asn) were treated with TAK475 at different concentrations for 6 days, and cell viability at different time points was detected by CCK8.


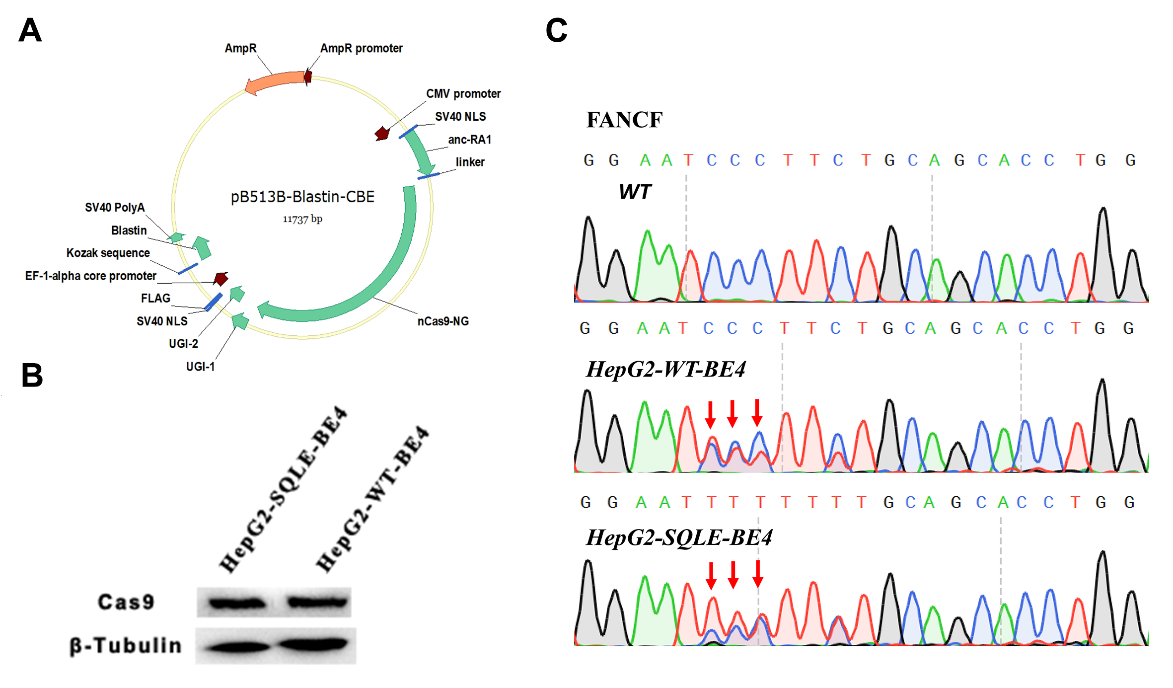


**Figure S3:** (A) Schematic of the pB513B-blastin-CBE plasmid. (B) Western blot analysis of Cas9 expression in HepG2-SQLE-BE4 and HepG2-WT-BE4 cells. (C) Sequence chromatogram of target regions in *FANCF*.


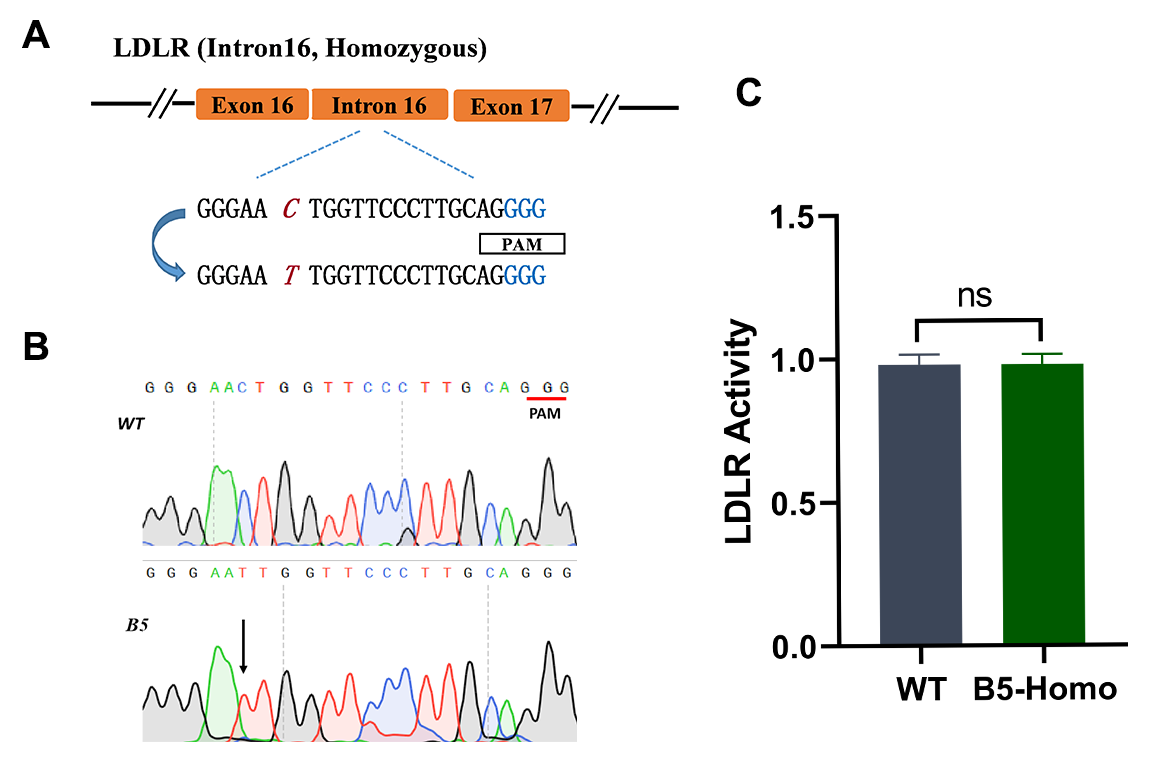


**Figure S4:** (A) The target sequence at the *LDLR* intron 16. The PAM sequence, sgRNA sequence, and substituted base are shown in blue, black, and red, respectively. (B) Sequence chromatogram of target regions in *LDLR* intron 16. (C) Comparison of LDLR activity between *LDLR* intron 16 homozygous mutant cells and WT cells.

| Name | Sequence (5’-3’) | PAM | Locus |
| --- | --- | --- | --- |
| SQLE-Target | **G-GT-TC-AGATTCC-T-T-A-C-CCTC-** | **TG** |  |
| SQLE-OTS1 | **G-GT-cC-AGATTCC-T-T-A-C-CCCtg** | **AG** | chr8: -79120409 |
| SQLE-OTS2 | **G-GT-TC-AGATTCC-T-T-ctC-CCaC-** | **TG** | chr15: -65096940 |
| SQLE-OTS3 | **G-cT-TCtAGATTCC-T-g-A-C-CCTC-** | **AG** | chr20: +6196304 |
| SQLE-OTS4 | **G-cT-TC-AGAATTCcT-g-A-C-CCTC-** | **AG** | chr20: +906435 |
| SQLE-OTS5 | **G-GT-tC-AGATTtC-T-T-A-a-CCTC-** | **TG** | chr14: +54011266 |
| SQLE-OTS6 | **G-GTcTC-AGATTCC-T-g-A-C-CCcC-** | **AG** | chr6: +136301375 |


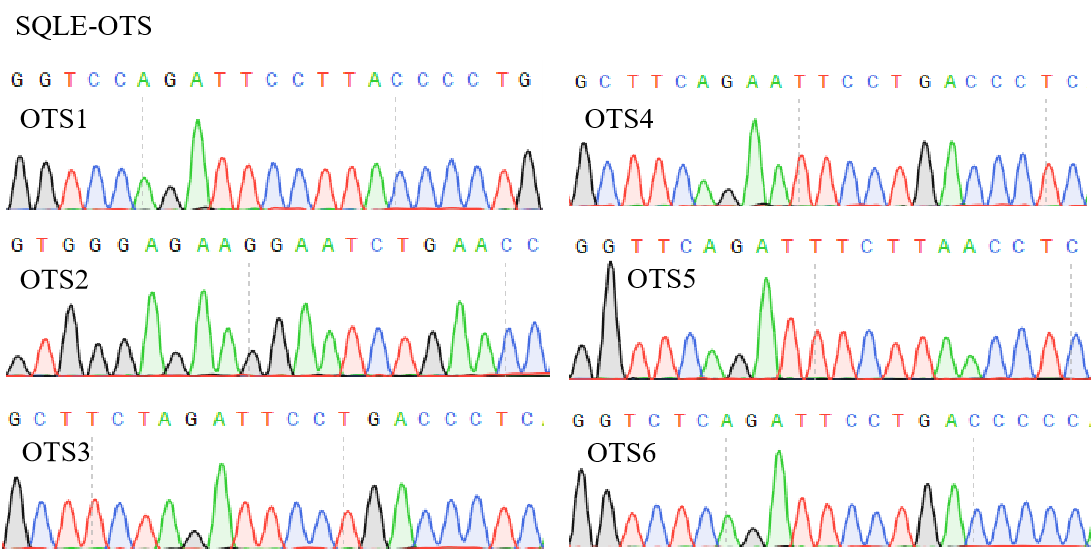


**Figure S5:** Off-target analysis of *SQLE* locus. OTS1~OTS6 represents 6 potential off-target sites. Base substitutions are shown in red.

| Name | Sequence (5’-3’) | PAM | Locus |
| --- | --- | --- | --- |
| LDLR-Target | **G-CAG-TCGGG-GTC-GT-TGTCGC** | **AG** |  |
| LDLR-OTS1 | **G-CAG-cCtGG-GTCtGT-TGTCGC** | **AG** | chr6: -8202318 |
| LDLR-OTS2 | **G-CAG-TCGGG-cTC-GT-TGcCaC** | **CG** | chr7: -32428062 |
| LDLR-OTS3 | **G-CAG-TCtGGgGTt-GT-TGTCaC** | **AG** | chr3: +101781466 |
| LDLR-OTS4 | **G-CgGcTCGGG-GTC-GT-TaTCaC** | **AG** | chr8: -87751394 |
| LDLR-OTS5 | **GaCAG-TgGGG-GTt-GT-TGTCcC** | **TG** | chr5: +30493076 |
| LDLR-OTS6 | **G-CAGcTCaGG-GgC-GT-TGTCGt** | **TG** | chr20: -61928241 |


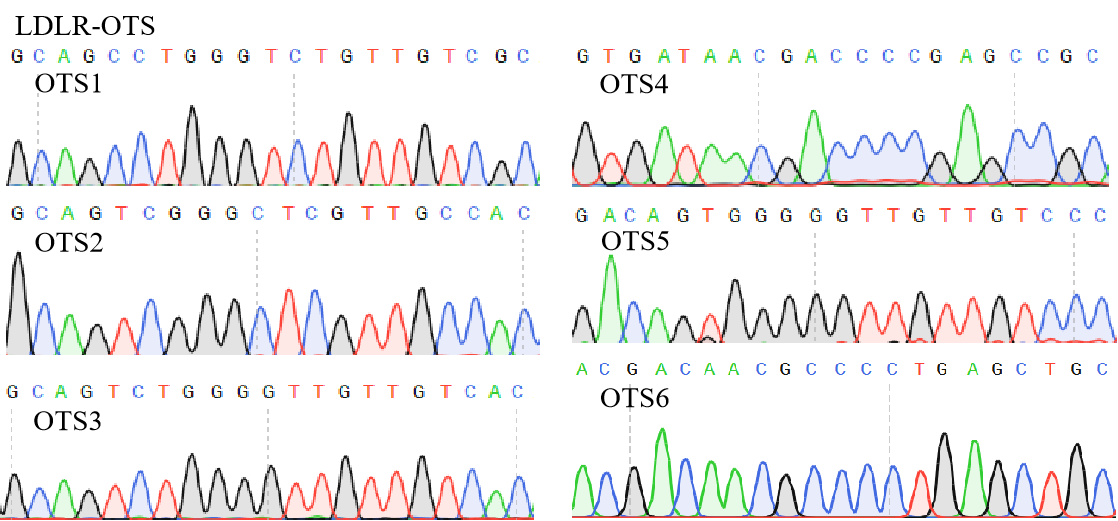


**Figure S6:** Off-target analysis of *LDLR* locus. OTS1~OTS6 represents 6 potential off-target sites. Base substitutions are shown in red. For Off-target site OTS5, there are nested peaks at this site on the human wild-type genome, not because of off-target effects.

| Name | Sequence (5’-3’) | PAM | Locus |
| --- | --- | --- | --- |
| LDLR-B1-Target | **-C-GAAACTCC-TC-CTCT-T-GCAG** | TG |  |
| LDLR-B1-OTS1 | **-aaGAAACTCC-TCt-TCT-T-GCAG** | **AG** | chr20: -51877111 |
| LDLR-B1-OTS2 | **-CtGAAACTCC-TCt-TCc-T-GCAG** | **CG** | chr7: +1529827 |
| LDLR-B1-OTS3 | **-C-cAAACTCCaTC-CTCT-T-GCAc** | **AG** | chr2:-145757964 |
| LDLR-B1-OTS4 | **-C-cAAACTCC-TC-CTCT-T-GCAc** | **TG** | chr6:-33870395 |
| LDLR-B1-OTS5 | **-t-GAcACTCtcTC-CTCT-T-GCAG** | **GG** | chr17: -7078111 |
| LDLR-B1-OTS6 | **-C-cAAAgTCC-TC-aCTC-TtGCAG** | **GG** | chr16: +87403696 |


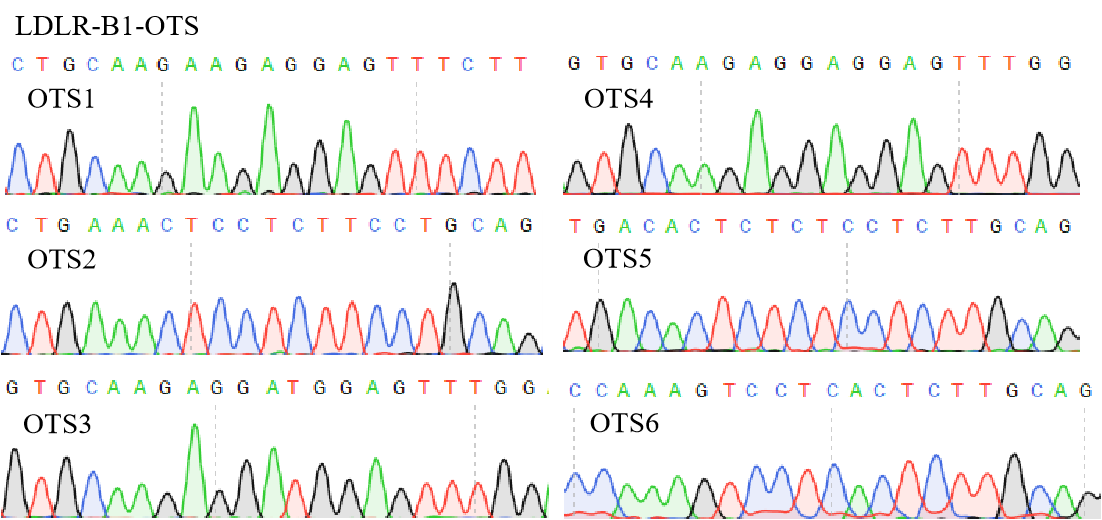


**Figure S7:** Off-target analysis of *LDLR* promoter locus. OTS1~OTS6 represents 6 potential off-target sites. Base substitutions are shown in red.

| Name | Sequence (5’-3’) | PAM | Locus |
| --- | --- | --- | --- |
| LDLR-B2-Target | **-ACTCGCTTAAACCCGGGAGG-** | **CG** |  |
| LDLR-B2-OTS1 | **gAaTCGCTTAAACCCGGGAGG-** | **TG** | chr5: -147480807 |
| LDLR-B2-OTS2 | **-ACTCGCTTgAACCCGGGAGGc** | **AG** | chr1: -31418459 |
| LDLR-B2-OTS3 | **-AaTCGCTTAAACCCaGGAGG-** | **CG** | chr8: -5131849 |
| LDLR-B2-OTS4 | **-ACTCaCTTgAACCCGGGAGG-** | **TG** | chr8: +73760778 |


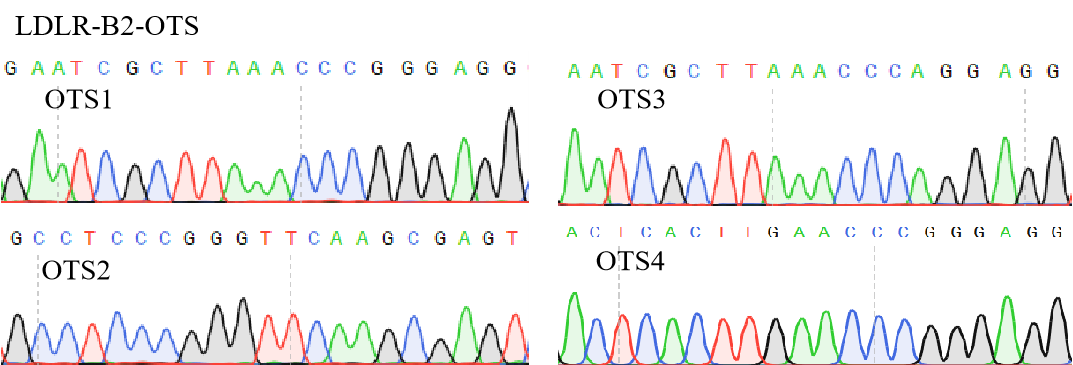


**Figure S8:** Off-target analysis of *LDLR* intron 1 locus. OTS1~OTS4 represents 4 potential off-target sites. Base substitutions are shown in red.

| Name | Sequence (5’-3’) | PAM | Locus |
| --- | --- | --- | --- |
| LDLR-B3-Target | **G-G-TCTC-C-GGTG-AAGAGCTGA** | **CG** |  |
| LDLR-B3-OTS1 | **G-GcTCTC-C-aGTG-AgGAGCTGA** | **GG** | chr5: +6468532 |
| LDLR-B3-OTS2 | **G-G-TCTC-CaGGTG-AAGAGgTGg** | **AG** | chr20: -19998902 |
| LDLR-B3-OTS3 | **G-G-TCTC-a-GtTGcAAGAGCTGA** | **GG** | chr2: +64244728 |
| LDLR-B3-OTS4 | **G-G-TCTC-C-GaTG-AAGgGCTGA** | **TG** | chr10: -71180577 |
| LDLR-B3-OTS5 | **t-G-TCTC-a-GGTGcAAGAGCTGA** | **CG** | chr13: -34002349 |
| LDLR-B3-OTS6 | **G-G-TCTC-CcaGTG-AcGAGCTGA** | **GG** | chr10: +132617149 |


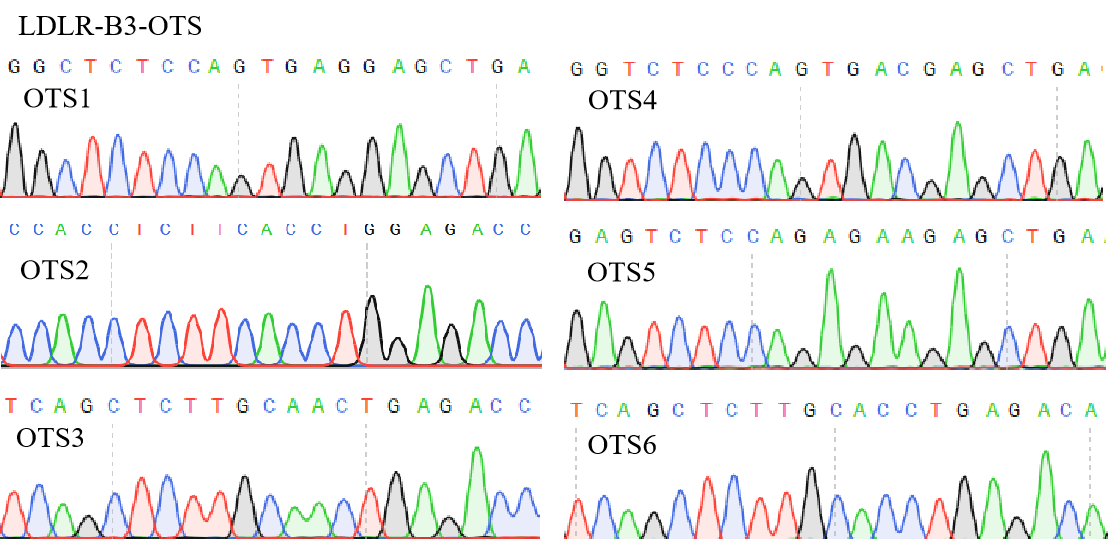


**Figure S9**: Off-target analysis of *LDLR* antisense RNA locus. OTS1~OTS6 represents 6 potential off-target sites. Base substitutions are shown in red.

| Name | Sequence (5’-3’) | PAM | Locus |
| --- | --- | --- | --- |
| LDLR-B4-Target | **-AG-TGGCC-GCCT-CTA-CTG-GGT** | **TG** |  |
| LDLR-B4-OTS1 | **-gG-aGGCCgGCCT-CTA-CTG-GGT** | **GG** | chr2: -239099822 |
| LDLR-B4-OTS2 | **-AG-TGcCt-GCCT-CTAaCTG-GGT** | **CG** | chr4: +147194813 |
| LDLR-B4-OTS3 | **-AG-TGGCC-GaCT-CTt-CTGcGGT** | **GG** | chr16: +88883640 |
| LDLR-B4-OTS4 | **-AG-TGGtCaGCCT-CTt-CTG-GGT** | **GG** | chr9: +129181237 |
| LDLR-B4-OTS5 | **-gG-TGGCC-GCCTgCTA-CTG-GGT** | **GG** | chr9: -134249055 |
| LDLR-B4-OTS6 | **-AG-TAGgCaGCCT-CTA-gTG-GGT** | **TG** | chr3: -169602571 |


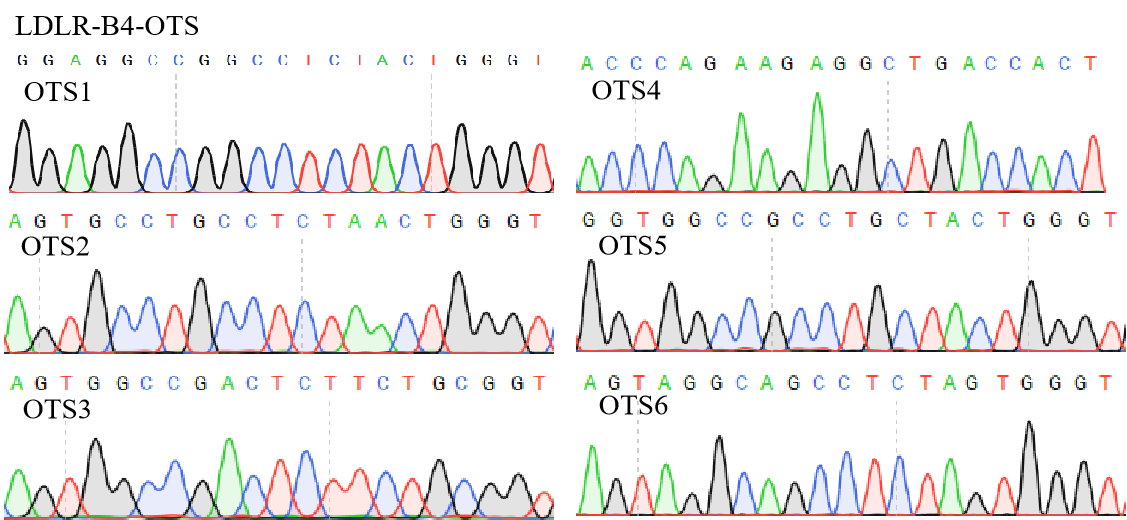


**Figure S10:** Off-target analysis of *LDLR* exon 12 locus. OTS1~OTS6 represents 6 potential off-target sites. Base substitutions are shown in red.

| Name | Sequence (5’-3’) | PAM | Locus |
| --- | --- | --- | --- |
| LDLR-B5-Target | **GGG-A-ACT-G-G-TTC-CCT-TGC-AG** | **GG** |  |
| LDLR-B5-OTS1 | **cGG-AtACT-G-G-TTt-CCT-TGC-AG** | **GG** | chr8: -115745416 |
| LDLR-B5-OTS2 | **GGG-A-ACT-GtG-TTC-aCT-TcC-AG** | **GG** | chr20: -60693200 |
| LDLR-B5-OTS3 | **GGG-A-Atc-G-G-TTCtCCT-TGC-AG** | **GG** | chr7: -156260353 |
| LDLR-B5-OTS4 | **aGG-A-ACT-G-G-TTC-CCTcTGC-Ac** | **AG** | chr19: -51143571 |
| LDLR-B5-OTS5 | **GGGcA-ACT-G-c-TTC-CaT-TGC-AG** | **GG** | chr18: +57044451 |
| LDLR-B5-OTS6 | **GGG-A-ACT-G-G-TaC-CCT-TGCctG** | **CG** | chr11: -3984992 |


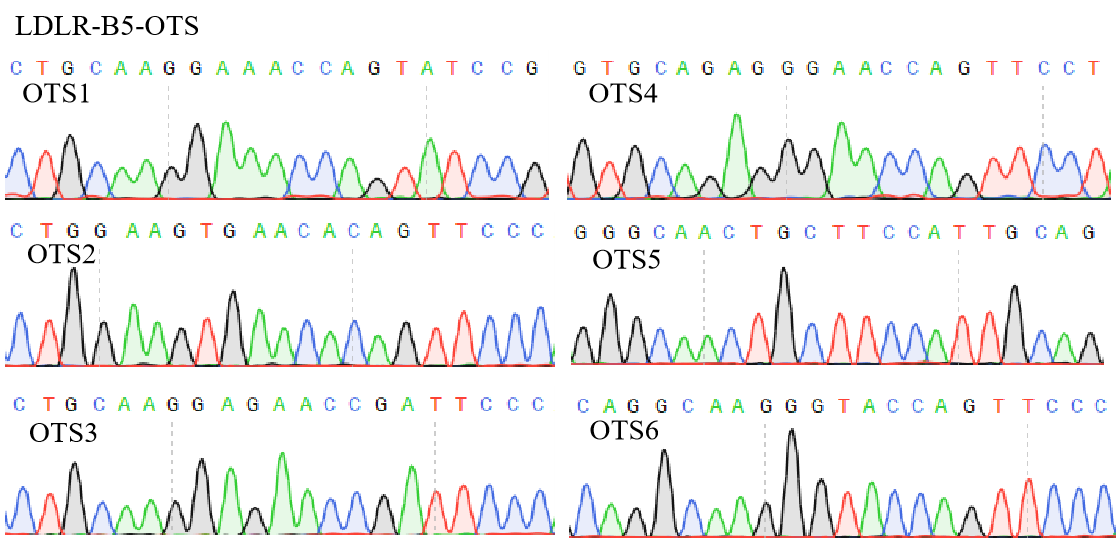


**Figure S11:** Off-target analysis of *LDLR* intron 16 locus. OTS1~OTS6 represents 6 potential off-target sites. Base substitutions are shown in red.


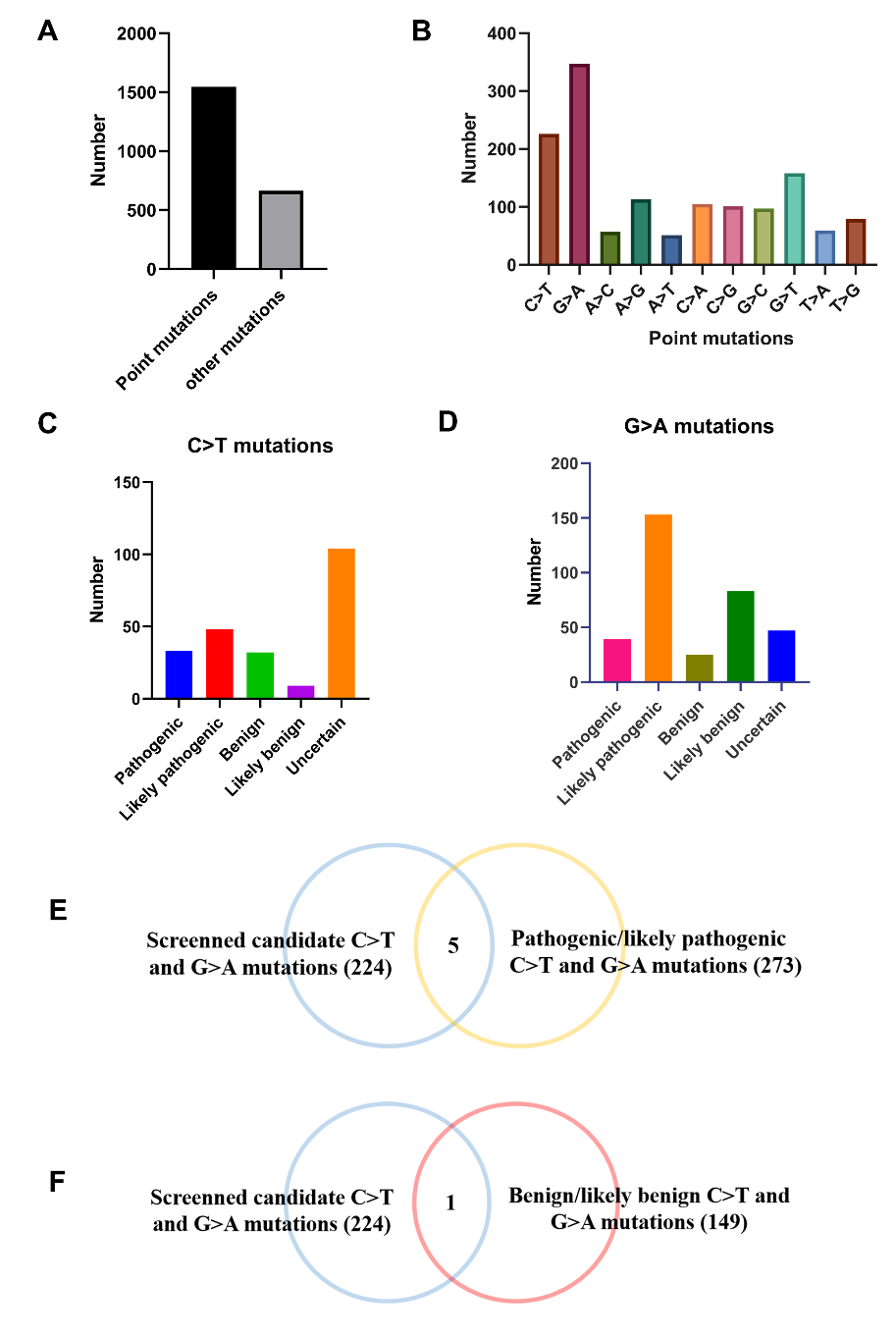


**Figure S12:** Comparative analysis of the screened candidate mutations and LOVD database mutations. (A) The number of point mutations and other mutations in LOVD database. (B) Classification of point mutations in LOVD database. (C) Clinical classification of C>T mutations. (D) Clinical classification of G>A mutations. (E) Intersection of screened candidate point mutations and pathogenic/likely pathogenic point mutations in LOVD database. (F) Intersection of screened candidate point mutations and benign/likely benign point mutations in LOVD database.


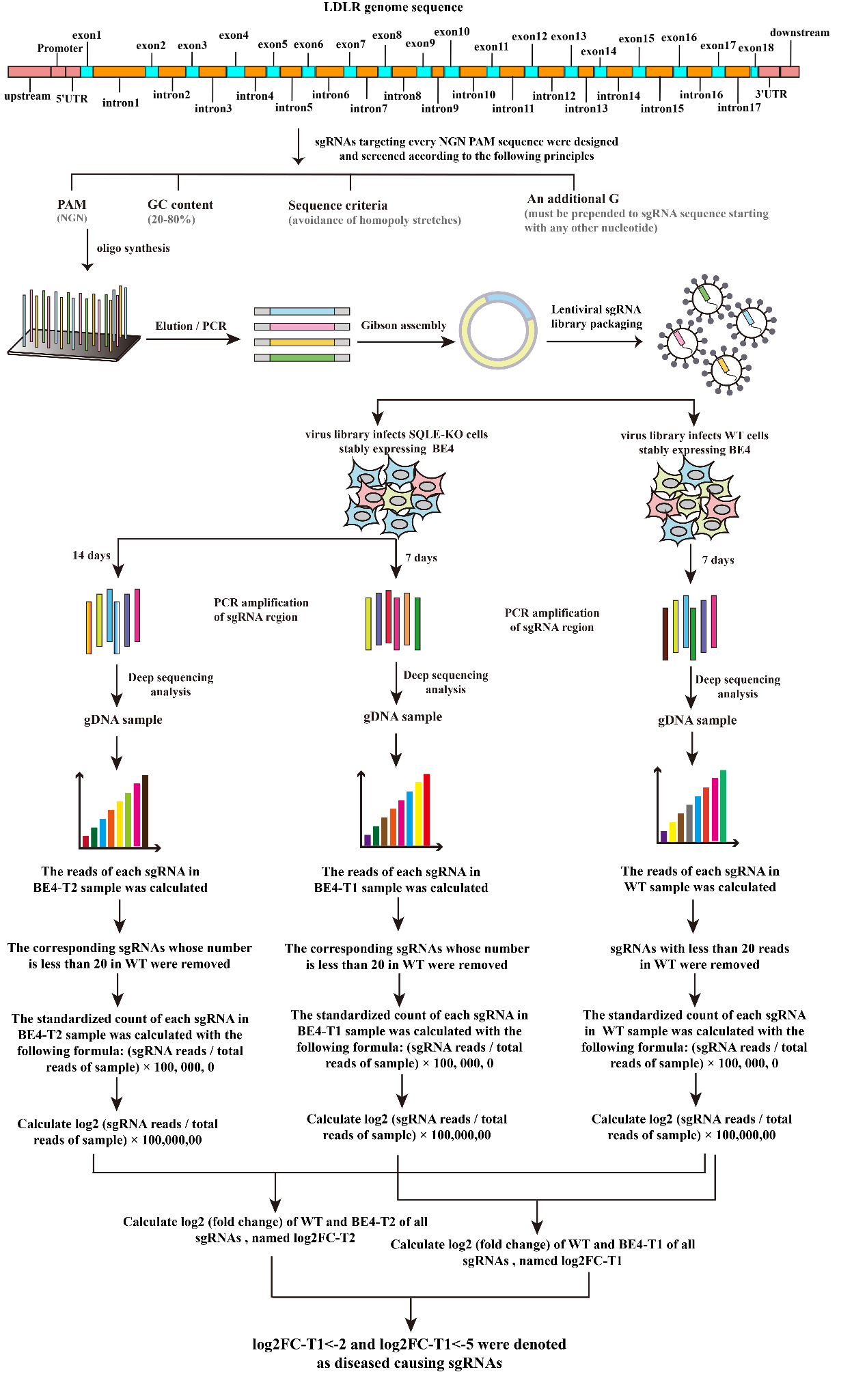


**Figure S13:** The overall schematic diagram of the whole experimental procedure.

Table S1: Statistical table for screening individual cell colonies

| Transfected cell type | Transfection method | plasmid | Total number of screened clones | The number of cells of each genotype |
| --- | --- | --- | --- | --- |
| Wild-type HepG2 | Electrotransfection | SQLE-specific sgRNA expression plasmid and NG-AncBE4max plasmid | 40 | Homozygous: 5  Heterozygous: 18  Wild-type: 17 |
| HepG2-SQLE-KO | Electrotransfection | *LDLR* pathogenic-specific sgRNA expression plasmid and NG-AncBE4max plasmid | 40 | Homozygous: 0  Heterozygous: 12  Wild-type: 28 |
| Heterozygous HepG2-SQLE-KO cells with *LDLR* pathogenic mutations | Electrotransfection | *LDLR* pathogenic-specific sgRNA expression plasmid and NG-AncBE4max plasmid | 40 | Homozygous: 0  Heterozygous: 40  Wild-type: 0 |
| Wild-type HepG2 | Electrotransfection | *LDLR* pathogenic-specific sgRNA expression plasmid and NG-AncBE4max plasmid | 40 | Homozygous: 3  Heterozygous: 15  Wild-type: 22 |

Table S2: The primer sequence used in this study

| Name | Sequence (5’-3’) |
| --- | --- |
| LDLR-lenti-F | GGACTATCATATGCTTACCGTAAC |
| LDLR-lenti-R | CAAGTTGATAACGGACTAGCC |
| LDLR-SQLE-F | CTGAAGTTGAAGTGGAATTACAGAC |
| LDLR-SQLE-R | GTCAAAGGAGAACTCTTCATAGTTC |
| LDLR-F | GTTGGGAGACTTCACACGGT |
| LDLR-R | ACCTTCCAAACAGTGATTTCCCA |
| LDLR-B1-F | CAGAGAGGACAATGGCATTAGG |
| LDLR-B1-R | CTGGAGCAAGCCTTACCTGC |
| LDLR-B2-F | GACACAGCAGGTCGTGATCC |
| LDLR-B2-R | CTCGCCCCCAGAAGGACTTA |
| LDLR-B3-F | CAGAGAGGACAATGGCATTAGG |
| LDLR-B3-R | CTGGAGCAAGCCTTACCTGC |
| LDLR-B4-F | GTTCAGGCTCACATGTGGTTG |
| LDLR-B4-R | CTGCGTTCATCTTGGCTTGAG |
| LDLR-B5-F | GTCAGATGGAGGATGTGGAG |
| LDLR-B5-R | GAGATGTGATGGTAACCGAG |
| LDLR-RT-F | CTTCGAGTTCCACTGCCTAAG |
| LDLR-RT-R | CCAACTTCATCGCTCATGTC |
| HMGCR-RT-F | CTCAGTTCCAACTCACAGGATG |
| HMGCR-RT-R | CACGAAGTAGTTGGCAAGAACTG |
| SREBP2-RT-F | GCAACAACAGACGGTAATGATC |
| SREBP2-RT-R | CTGTACTGTCTGCACCTGCTG |
| NPC2-RT-F | GACAGTCTTACAGCGTCAATGTC |
| NPC2-RT-R | GTCATCCTGAAGTTGCCACTC |
| CI-MPR-RT-F | GTATGCACGACTTGAAGACACG |
| CI-MPR-RT-R | GTGCACACATTCTGTTGCAGTTAC |
